# LncRNA Gas5 directs SUV39H2 to establish heterochromatin and maintain genome stability

**DOI:** 10.1093/procel/pwag039

**Published:** 2026-06-23

**Authors:** Juntao Liu, Jiani Yang, Wen Ye, Dandan Zhang, Nan Su, Hong Wang, Yanping Zhang, Shaorong Gao, Lan Kang

**Affiliations:** Institute for Regenerative Medicine, Shanghai East Hospital, Shanghai Institute of Stem Cell Research and Clinical Translation, Shanghai Key Laboratory of Signaling and Disease Research, Frontier Science Center for Stem Cell Research, School of Life Sciences and Technology, Tongji University, Shanghai 200092, China; Institute for Regenerative Medicine, Shanghai East Hospital, Shanghai Institute of Stem Cell Research and Clinical Translation, Shanghai Key Laboratory of Signaling and Disease Research, Frontier Science Center for Stem Cell Research, School of Life Sciences and Technology, Tongji University, Shanghai 200092, China; Institute for Regenerative Medicine, Shanghai East Hospital, Shanghai Institute of Stem Cell Research and Clinical Translation, Shanghai Key Laboratory of Signaling and Disease Research, Frontier Science Center for Stem Cell Research, School of Life Sciences and Technology, Tongji University, Shanghai 200092, China; Institute for Regenerative Medicine, Shanghai East Hospital, Shanghai Institute of Stem Cell Research and Clinical Translation, Shanghai Key Laboratory of Signaling and Disease Research, Frontier Science Center for Stem Cell Research, School of Life Sciences and Technology, Tongji University, Shanghai 200092, China; Institute for Regenerative Medicine, Shanghai East Hospital, Shanghai Institute of Stem Cell Research and Clinical Translation, Shanghai Key Laboratory of Signaling and Disease Research, Frontier Science Center for Stem Cell Research, School of Life Sciences and Technology, Tongji University, Shanghai 200092, China; Clinical and Translation Research Center of Shanghai First Maternity & Infant Hospital, School of Life Sciences and Technology, Tongji University, Shanghai 200092, China; Clinical and Translation Research Center of Shanghai First Maternity & Infant Hospital, School of Life Sciences and Technology, Tongji University, Shanghai 200092, China; Frontier Science Center for Stem Cell Research, Tongji University, Shanghai 200092, China; Clinical and Translation Research Center of Shanghai First Maternity & Infant Hospital, School of Life Sciences and Technology, Tongji University, Shanghai 200092, China; Frontier Science Center for Stem Cell Research, Tongji University, Shanghai 200092, China; Institute for Regenerative Medicine, Shanghai East Hospital, Shanghai Institute of Stem Cell Research and Clinical Translation, Shanghai Key Laboratory of Signaling and Disease Research, Frontier Science Center for Stem Cell Research, School of Life Sciences and Technology, Tongji University, Shanghai 200092, China; Frontier Science Center for Stem Cell Research, Tongji University, Shanghai 200092, China

**Keywords:** heterochromatin, H3K9me3, SUV39H2, Gas5, genome stability

## Abstract

Constitutive heterochromatin, characterized by histone H3 lysine 9 trimethylation (H3K9me3), is essential for genome stability, pluripotency, and developmental fidelity. While the SUV39H histone methyltransferases catalyze H3K9me3 deposition, their locus-specific targeting mechanisms remain unclear. Here, via RNA depletion or RNA-binding affinity mutation, we establish that RNA binding is indispensable for SUV39H2 recruitment to chromatin and H3K9me3 maintenance in mouse embryonic stem cells (ESCs). Through RNA immunoprecipitation sequencing (RIP-Seq) and functional validation, we identify the long non-coding RNA Gas5 as a specific SUV39H2 interactor. Depletion of Gas5 or disruption of the SUV39H2–RNA interaction leads to the loss of self-renewal capacity of ESCs, abolishes H3K9me3 enrichment, along with profound genomic instability, mitotic errors, and γH2AX foci accumulation. These findings reveal a critical lncRNA-dependent mechanism governing the SUV39H2–H3K9me3 axis, which directly couples RNA metabolism to the preservation of pluripotency and genome integrity in stem cells.

## Introduction

The eukaryotic genome is hierarchically organized into chromatin, which is functionally partitioned into transcriptionally active euchromatin and repressive heterochromatin (Heitz, 1928; Solovei and Mirny, 2024). Heterochromatin plays critical roles in silencing repetitive elements, preventing aberrant recombination, and ensuring faithful chromosome segregation during mitosis (Bulut-Karslioglu et al., 2014; Escobar et al., 2019; Grewal and Moazed, 2003; Peters et al., 2001; Tachibana et al., 2007). Nucleosomes within heterochromatin carry histone post-translational modifications that vary dynamically during development, contributing to cell fate determination and developmental processes (Allshire and Madhani, 2018; Janssen et al., 2018; Nicetto and Zaret, 2019). Disruption of heterochromatic structures is frequently associated with developmental defects (Checchi and Engebrecht, 2011; Dodge et al., 2004; Tachibana et al., 2002; Zeller et al., 2016).

The hallmark of constitutive heterochromatin is trimethylation of histone H3 at lysine 9 (H3K9me3) (Rea et al., 2000). This repressive mark serves as a high-affinity binding platform for Heterochromatin Protein 1 (HP1), promoting chromatin compaction that restricts transcription factor accessibility (Bannister et al., 2001; Lachner et al., 2001; Nakayam et al., 2001). In mammals, the SUV39H family (SUV39H1 and SUV39H2) of histone methyltransferases is primarily responsible for catalyzing H3K9me3 deposition (Lachner et al., 2001; Nakayam et al., 2001; Rea et al., 2000). While the catalytic activity of these enzymes is well-characterized, the precise mechanisms governing their locus-specific targeting remain a critical area of investigation.

Emerging evidence underscores the pivotal role of non-coding RNAs (ncRNAs) in directing chromatin-modifying complexes to their genomic target sites. The RNA-mediated heterochromatin assembly paradigm was first established in *Schizosaccharomyces pombe* (*S. pombe*), where nascent transcripts and the RNA interference (RNAi) machinery recruit the sole H3K9 methyltransferase, Clr4, to centromeric repeats (Hall et al., 2002; Verdel et al., 2004; Volpe et al., 2002). In mammals, diverse RNAs—including long non-coding RNAs (lncRNAs)—have been reported to associate with chromatin modifiers (Camacho et al., 2017; Johnson et al., 2017), but the functional relevance of these interactions for H3K9me3 maintenance remains poorly understood.

In this study, we demonstrate that RNA binding is a prerequisite for SUV39H2 recruitment to chromatin and H3K9me3 maintenance in embryonic stem cells (ESCs). We identified the lncRNA Gas5 as a specific partner of SUV39H2. Depletion of Gas5 or disruption of the SUV39H2-RNA interaction results in loss of H3K9me3 and severe defects in genomic integrity, along with loss of self-renewal capacity. Our findings reveal a critical lncRNA-mediated mechanism governing the SUV39H2-H3K9me3 axis, linking RNA metabolism to the preservation of ESC identity and genome stability.

## Results

### Interaction with RNA is required for SUV39H2 recruitment to chromatin

SUV39H2 has been reported to bind diverse RNAs, including lncRNAs and mRNAs (Camacho et al., 2017). If any of these RNAs participate in the SUV39H2–chromatin interactions, recruitment would be sensitive to RNA depletion. To characterize RNA-dependent chromatin localization, we employed RNA-dependent chromatin immunoprecipitation (ChIP) coupled with deep sequencing (rChIP–seq) (Long et al., 2020), in which RNase A was added during the immunoprecipitation step (Fig. 1A). rChIP–seq revealed a dramatic loss of SUV39H2 chromatin localization upon RNase A treatment (Figs. 1B, 1C and S1A). In contrast, RPB1, a well-characterized DNA-binding subunit of RNA polymerase II with DNA-binding activity independent of RNA, exhibited chromatin localizations unaffected by RNase A treatment (Figs. 1B, S1A  and S1B). The RNase A-induced loss of SUV39H2 occupancy at canonical target sites was confirmed by ChIP coupled with quantitative PCR (ChIP-qPCR) (Fig. 1D).

**Figure 1.**
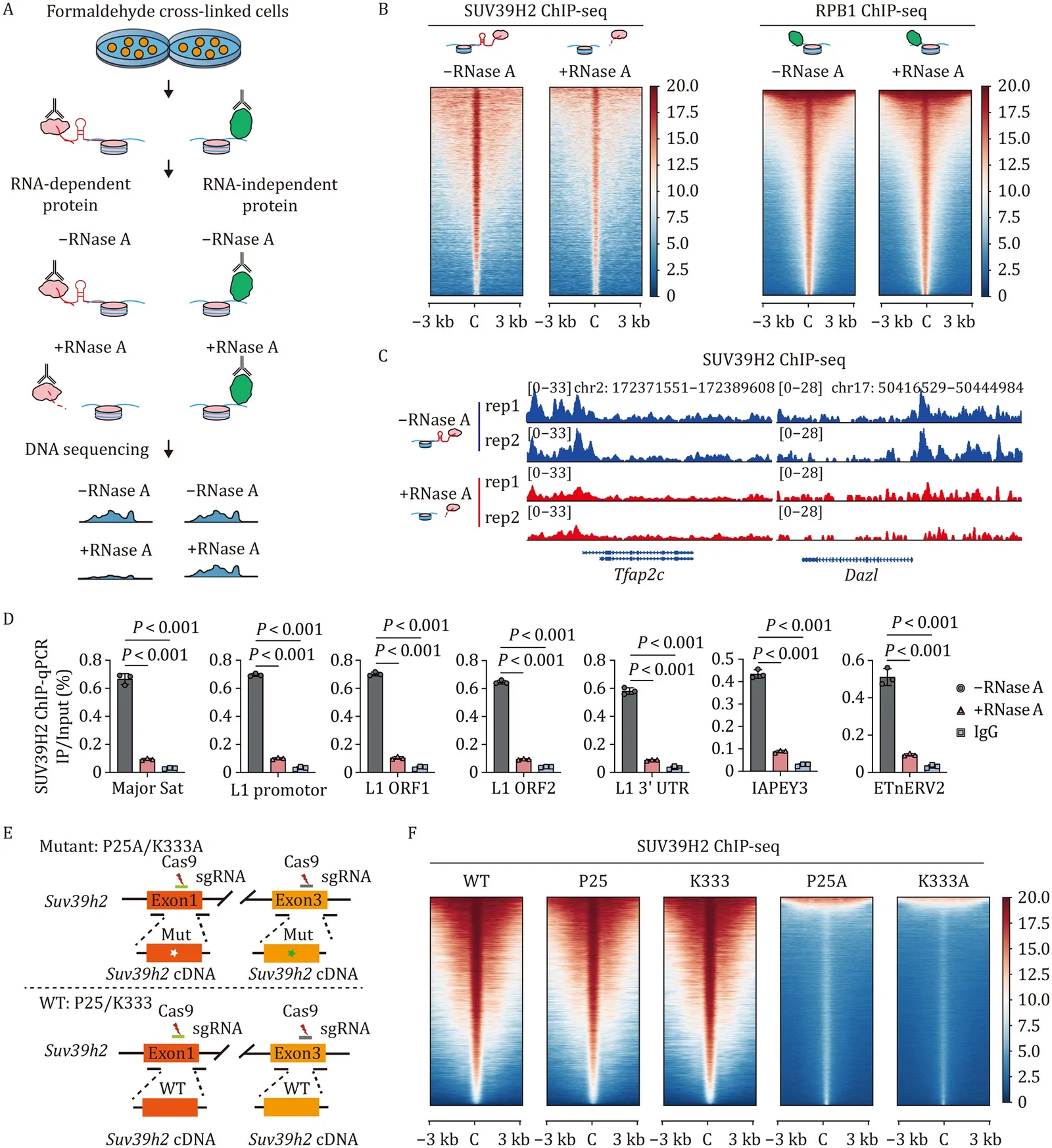
**RNA is essential for SUV39H2 chromatin occupancy**. (A) Schematic of the RNA-dependent ChIP (rChIP) experiment design. RNase A is added (+RNase A) or omitted (−RNase A) during the immunoprecipitation step of standard ChIP procedure. RNA-dependent protein–chromatin interactions would be disrupted by RNase A treatment, which degrades the RNA linkage (red strand) and releases the protein (pink square) from chromatin. (B) Heat maps of rChIP–seq data for SUV39H2 and RPB1 in ESCs. The scale below indicates the peak center (C) and the 3 kilobases (3 kb) upstream/downstream of the peak. Color intensity represents fold change (IP/Input). (C) Representative genomic tracks of rChIP–seq experiments. The *Tfap2c* and *Dazl* loci are shown in SUV39H2 rChIP–seq data. The y-axis shows fold change (IP/Input), with −RNase A (blue) and +RNase A (red). (D) ChIP-qPCR analysis of SUV39H2 binding at target regions gene loci in +RNase A versus −RNase A samples (mean ± SEM; *n *= 3 biological replicates). (E) Schematic of CRISPR-Cas9-mediated generation of WT (P25/K333) and Mut (P25A/K333A) SUV39H2 ESC lines. (F) Heat maps of ChIP–seq data for WT, WT (P25/K333), and Mut (P25A/K333A) in ESCs. The scale below indicates the peak center (C) and 3 kb upstream/downstream of the peak. Color intensity represents fold change (IP/Input).

Nascent RNA has been reported to function as a feedback regulator or a scaffold that recruits silencing machinery to heterochromatin assembly (Henninger and Young, 2024). To test the functional requirement of nascent RNA for SUV39H2 localization, we treated cells with Actinomycin D, an inhibitor of RNA polymerase II, followed by SUV39H2 ChIP–seq analysis. Similar to RNase A treatment, Actinomycin D induced a global loss of SUV39H2 chromatin occupancy (Fig. S1C–E).

To provide more specific evidence, we generated endogenous SUV39H2 RNA-binding-defective point mutants. Using the online tool catRAPID (Armaos et al., 2021) and RBPDB (Cook et al., 2011) to predict protein-RNA binding affinity, we identified key surface residues of SUV39H2 critical for RNA interaction. We independently introduced P25A and K333A mutations into the endogenous SUV39H2 locus in ESCs via CRISPR-Cas9-mediated homology-directed repair (HDR) (Fig. 1E), generating two distinct RNA-binding deficient mutant cell lines (hereafter collectively referred to as “Mut (P25A/K333A)”). To mitigate potential off-target effects or artifacts from genome editing, we generated isogenic wild-type (WT) SUV39H2 cell lines alongside the mutant SUV39H2 cell lines using the same CRISPR guide RNA, but synonymous mutation donor DNA (Fig. 1E). The protein levels of genome-edited WT (P25/K333) and mutant (P25A/K333A) SUV39H2 were comparable (Fig. S1F and S1G), with proper nuclear localization (Fig. S1G). To assess the RNA-binding capacity of SUV39H2, we selected RNAs previously reported to interact with SUV39H2 *in vitro* (Camacho et al., 2017). As native RIP-qPCR showed, while WT SUV39H2 showed robust affinity for RNAs, this association was severely impaired in the mutant cell lines (Fig. S1H). SUV39H2 ChIP-seq using these cell lines showed that, consistent with RNA depletion assays, the mutation in the RNA-binding site of SUV39H2 destroyed its localization at WT SUV39H2 high-occupancy sites (Figs. 1F,  S1I and S1J).

Collectively, these results strongly suggested that the interaction with RNAs is a prerequisite for proper recruitment of SUV39H2 to chromatin in ESCs.

### RNA-binding activity of SUV39H2 is crucial for global H3K9me3 deposition and cell identity maintenance

SUV39H2 is well known crucial for H3K9me3 deposition (Brumbaugh et al., 2019; Chen et al., 2013; Nicetto and Zaret, 2019; Soufi et al., 2012) and cell identity maintenance (Balan et al., 2021; Bulut-Karslioglu et al., 2014). Consistently, SUV39H2 depletion in ESCs resulted in loss of sharp-edged clonal morphology and a marked reduction of global H3K9me3 level (Fig. 2A–C). Similar phenotypes were observed in SUV39H2 RNA-binding mutants (P25A/K333A), albeit milder than in knockout (KO) cells (Figs. 2D, 2E and S2A), demonstrating that RNA-binding is required for SUV39H2 function and pluripotency maintenance. The reduction was specific to H3K9me2 and H3K9me3, with H3K9me1 levels unchanged (Fig. S2B). Furthermore, to exclude variability arising from genetic manipulation, we introduced exogenous WT, empty vector (EV), or mutant SUV39H2 into SUV39H2-depleted cells. Re-expression of WT SUV39H2 fully restored H3K9me3 levels, whereas both RNA-binding-defective mutants (P25A/K333A) failed (Figs. 2F, 2G and S2C). To complement these genetic approaches, we employed OTS186935, a specific inhibitor that selectively targets the catalytic center of SUV39H2 without impacting SUV39H1 (Vougiouklakis et al., 2018). With increasing concentrations of OTS186935, while SUV39H2 protein levels and RNA-binding activity remained unchanged, global H3K9me3 levels were significantly reduced (Figs. 2H, S2D and S2E).

**Figure 2.**
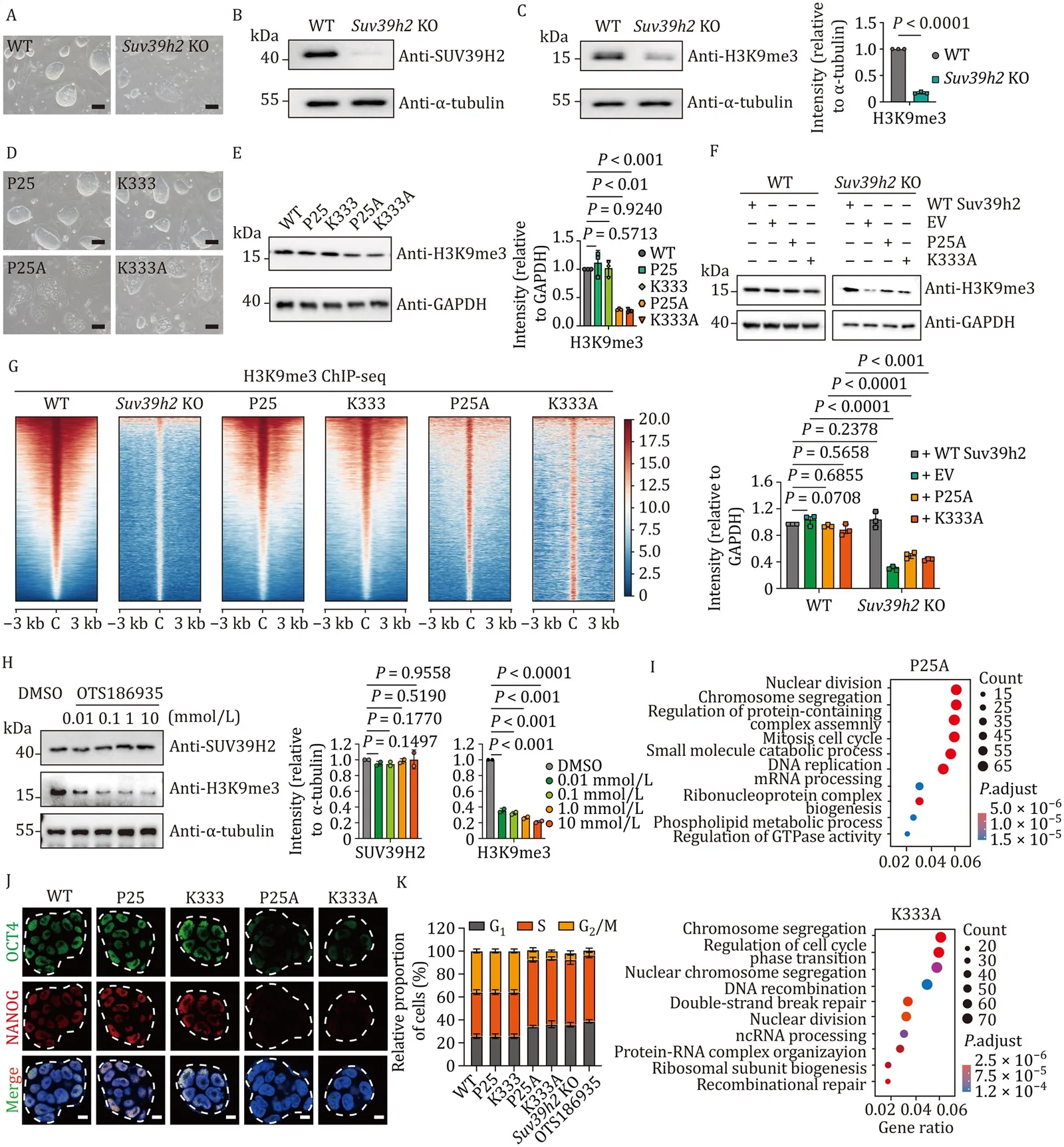
**RNA-binding activity of SUV39H2 is dispensable for maintaining global H3K9me3**. (A) Bright-field images of WT and *Suv39h2* KO ESC colonies. Scale bar: 100 µm. (B) Western blot of SUV39H2 in *Suv39h2* KO versus WT ESCs. α-Tubulin serves as a loading control. (C**)** Western blot of H3K9me3 in *Suv39h2* KO versus WT ESCs. Bar plots show quantifications of H3K9me3 intensity normalized to α-Tubulin. (D) Bright-field images of WT (P25/K333) and Mut (P25A/K333A) ESC colonies. Scale bar: 100 µm. (E) Western blot of H3K9me3 in WT, WT (P25/K333), and Mut (P25A/K333A) ESCs. Bar plots show quantifications of H3K9me3 intensity normalized to GAPDH. (F) Western blot of H3K9me3 in *Suv39h2* KO ESCs rescued with WT SUV39H2, empty vector (EV), or Mut (P25A/K333A). Bar plots show quantifications of H3K9me3 intensity normalized to GAPDH. (G) Heat maps of H3K9me3 ChIP–seq signals in *Suv39h2* KO ESCs rescued with WT SUV39H2 or Mut (P25A/K333A). Scale below indicates the peak center (C) and 3 kb upstream/downstream of the peak. Color intensity represents fold change (IP/Input). (H)Western blot of H3K9me3 in ESCs treated with DMSO or OTS186935. Bar plots show quantifications of H3K9me3 intensity normalized to α-Tubulin. (I) GO analysis of differentially expressed genes in Mut (P25A/K333A) compared to WT SUV39H2, where *p*-adjust represents Benjamini-Hochberg adjusted *P-*value. (J) Immunofluorescence staining of pluripotency-related protein OCT4 (green) and NANOG (red) in WT (EV), WT (P25/K333), and Mut (P25A/K333A) ESCs. Nuclei counterstained with DAPI (blue). Scale bars: 10 µm. (K) Cell cycle analysis of WT (EV), WT (P25/K333), and Mut (P25A/K333A) ESCs. Histograms show the percentage of cells in G_1_, S, or G_2_/M phase. Data in (C, E, F, H, and K) are shown as mean ± SEM (*n *= 3 independent experiments).

H3K9me3 is required to maintain the repressed state of cell type-specific genes (Brumbaugh et al., 2019; Chen et al., 2013; Nicetto and Zaret, 2019), which in turn preserves transcriptional programs and cell identity. RNA-seq analysis revealed 598 and 722 differentially expressed genes in the two RNA-binding-defective mutants, respectively (Fig. S2F). These genes were enriched in GO terms associated with chromosomal segregation and cell cycle (Fig. 2I). Phenotypic analysis further demonstrated that mutant cell lines closely resembled *Suv39h2* KO cells, exhibiting aberrant colony morphology, downregulated pluripotency factors, and S-phase cell cycle arrest (Fig. 2J and 2K). Collectively, these results establish that the RNA-binding activity of SUV39H2 is indispensable for maintaining transcriptional programs and cell identity in ESCs.

### Characterization of RNAs involved in SUV39H2 chromatin occupancy

To systematically characterize the RNA interactome mediating SUV39H2 chromatin binding and function, we adapted an RNA immunoprecipitation sequencing (RIP-seq) protocol inspired by Mili and Steitz (2004). Briefly, we generated vectors expressing 3×Flag- and biotin-tagged proteins (^FB^SUV39H2 and ^FB^GFP), which were transfected into ESCs (Fig. S3A). Cell lysates were prepared under native conditions (without UV-Cross-linking), followed by tandem purification using Flag and biotin antibodies. For comparative analysis, we expressed tagged versions of SUV39H1 and SETDB1—an abundant SET domain-containing H3K9 methyltransferase reported to bind RNA *in vitro* (Johnson et al., 2017; Scarola et al., 2015).

RIP-seq identified RNAs significantly enriched in ^FB^SUV39H2 immunoprecipitation relative to ^FB^GFP control (fold-change > 2, *P *< 0.01). Genomic annotation revealed that majority reads originated from exonic (∼53%), promoter (∼14.5%), and intronic (∼13.9%) regions (Fig. 3A). GO analysis highlighted enrichment of the bound mRNAs in nuclear processes, including mRNA processing, RNA splicing, chromatin remodeling, chromosome segregation, and mitotic cell cycle phase transition (Fig. 3B). Integrating the RIP-seq data with ESC RNA-seq profiles, we identified five candidate lncRNAs (Rmrp, Malat1, Gas5, Snhg12, and Neat1) (Fig. 3C and 3D), several of which have been implicated in widespread protein interactions (Hirata et al., 2015; Huang et al., 2022; West et al., 2014). These bindings were also supported by crosslinking-based immunoprecipitation sequencing (CLIP-seq) (Fig. 3D). RIP-qPCR in ESCs expressing WT and RNA-binding-deficient SUV39H2 further confirmed these specific interactions (Fig. S3B).

**Figure 3.**
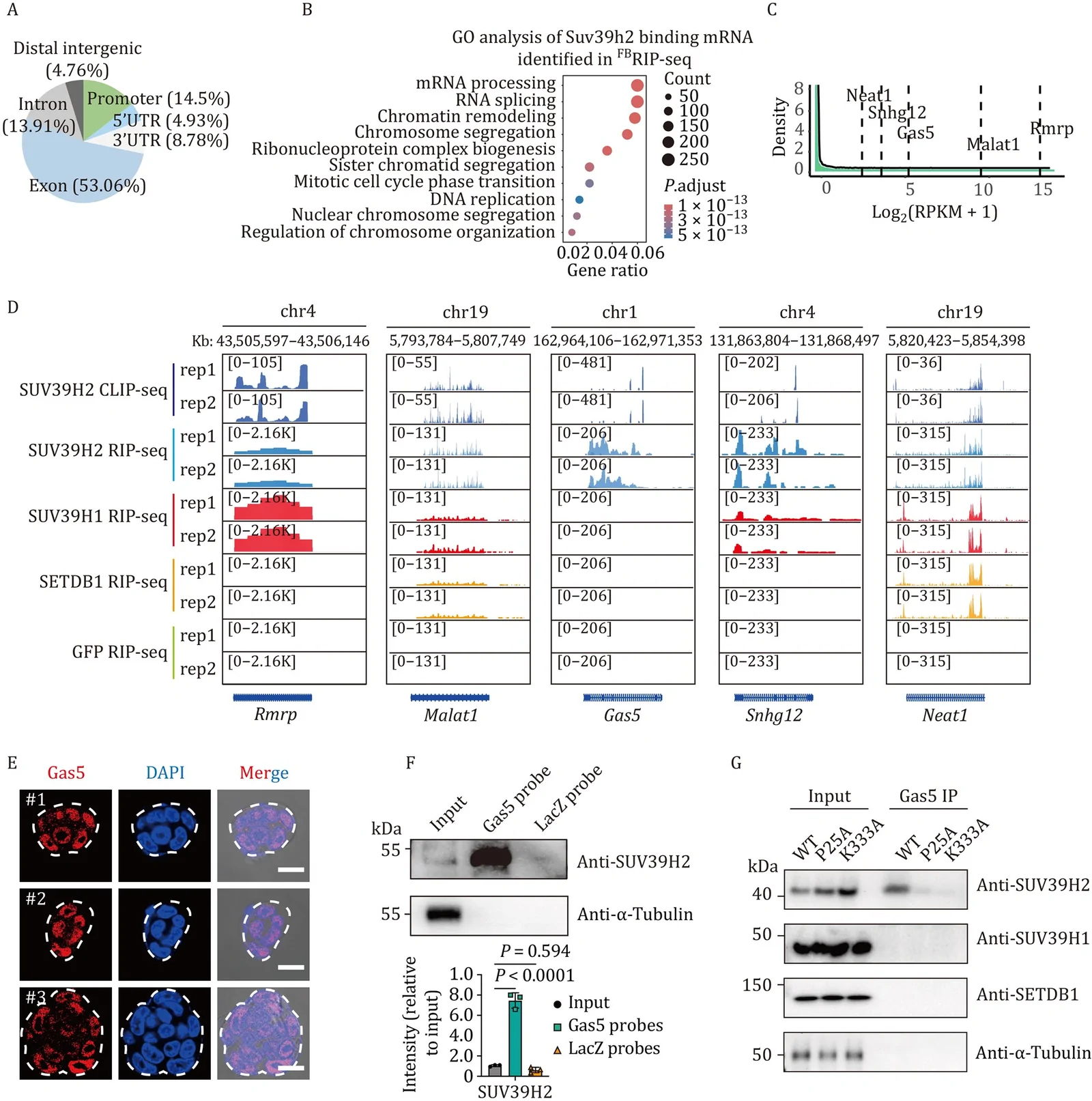
**Characterization of the RNA interactome involved in SUV39H2 chromatin occupancy**. (A) Pie chart annotating peaks identified by SUV39H2 RIP-seq. Peaks were classified into genomic features: promoters, exons, introns, 5′UTRs, 3′UTRs, and intergenic regions. UTR: untranslated region. (B) GO analysis of high-confidence SUV39H2-interacting mRNAs identified by RIP^FB^-seq (RNA Immunoprecipitation using FLAG-Biotin dual tags). SUV39H2 was fused to FLAG and biotin tags, followed by sequential immunoprecipitation with anti-FLAG and streptavidin bead enrichment. Significantly enriched biological processes are ranked by fold enrichment. (C) Expression ranking of lncRNAs prioritized through joint analysis of SUV39H2 RIP^FB^-seq and RNA-seq data. (D) Representative genomic tracks showing RIP^FB^-seq enrichment profiles at *Rmrp*, *Malat1*, *Gas5*, *Snhg12*, and *Neat1* loci in ESCs. Data compare RNA binding profiles of SUV39H2, SUV39H1, SETDB1, and GFP (negative control). (E) RNA FISH staining of Gas5 (red) in ESCs. Nuclei were counterstained with DAPI (blue). Representative images from three independent clones are presented. Scale bars: 20 μm. (F) *In vitro* RNA pull-down using biotinylated Gas5-specific probes or lacZ control probes, followed by western blot for SUV39H2. Bar plots show quantifications of SUV39H2 intensity normalized to Input. (G) *In vitro* RNA pull-down assay using biotinylated Gas5-specific probes in the lysates of WT and Mut (P25A/K333A) ESCs, followed by western blotting for SUV39H2, SUV39H1, and SETDB1. LacZ probe serves as a negative control. Data in F and G are shown as mean ± SEM (*n *= 3 independent experiments).

We reasoned that functionally relevant SUV39H2-interacting RNAs should exhibit nuclear enrichment. Quantification of nuclear/cytoplasmic ratios confirmed that Gas5 and Malat1 were selectively enriched in the nucleus, mirroring the localization of the well-characterized nuclear lncRNA Neat1 (Figs. 3E and S3C). Notably, Gas5 specifically associated with SUV39H2 but not SUV39H1 or SETDB1, despite high structural similarity in their SET domains (Figs. 3D and S3D).

On the other hand, we employed chromatin oligo-affinity precipitation (ChOP) using biotinylated antisense DNA probes targeting Gas5 to pull down its interacting proteins. Western blot revealed that SUV39H2 was efficiently retrieved by *Gas5* probes, whereas no enrichment was observed with negative control (lacZ) probes (Fig. 3F). RNA pull-down assays using *in vitro* transcribed biotinylated Gas5 RNA further confirmed specific capture of WT SUV39H2, but not SUV39H1, SETDB1, or RNA-binding-deficient SUV39H2 (Fig. 3G).

Collectively, these results demonstrate that SUV39H2 directly and specifically binds RNAs. The specificity of Gas5 binding suggests a potential mechanism for guiding SUV39H2 to its genomic binding loci.

### LncRNA Gas5 is essential for H3K9me3 heterochromatin and SUV39H2 chromatin occupancy

To investigate the function of Gas5, we generated *Gas5* KO cells using a CRISPR-Cas9-mediated inducible ablation system targeting the exonic regions of *Gas5*. This approach achieved robust depletion of Gas5 in ESCs (Fig. S4A and S4B). *Gas5*-ablated ESCs exhibited a marked loss of typical dome-shaped colony morphology (Fig. 4A), accompanied by the downregulation of pluripotency markers and a concomitant upregulation of germ-layer-specific genes, especially the endoderm markers, leading to biased differentiation tendency as assessed by embryoid body (EB) differentiation assay (Figs. 4B, S4C and S4D). Furthermore, cell proliferation was significantly impaired, attributed to persistent S-phase cell cycle arrest (Fig. 4C and 4D). Notably, these cellular defects phenocopied those observed upon the loss of the RNA-binding activity of SUV39H2, suggesting a functional nexus between the SUV39H2 and Gas5.

**Figure 4.**
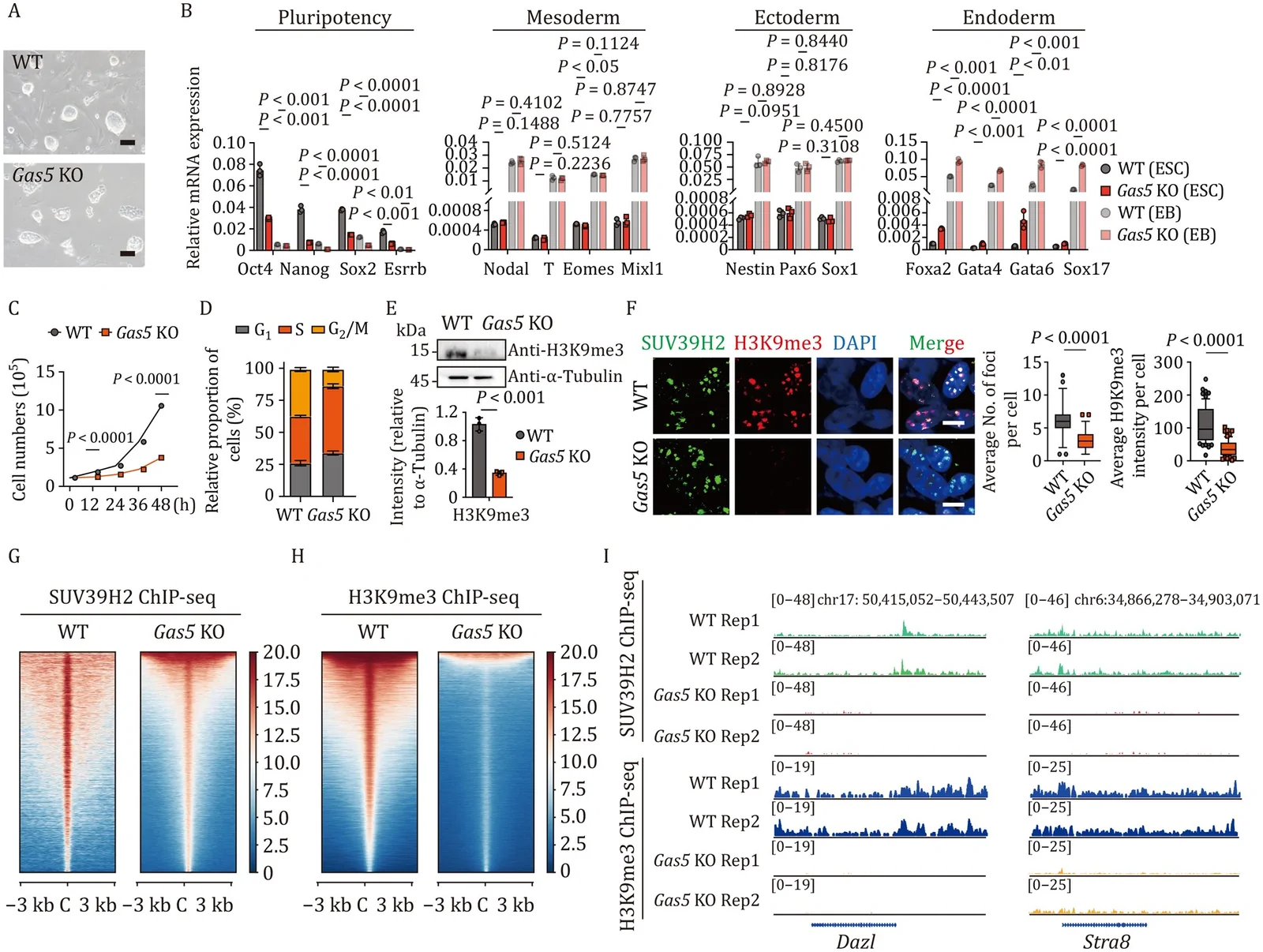
**LncRNA Gas5 is essential for H3K9me3 heterochromatin and SUV39H2 chromatin occupancy**. (A) Bright-field images of WT and *Gas5* KO ESC colonies. Scale bar: 100 µm. (B) qPCR analysis of pluripotency-, ectoderm-, mesoderm-, and endoderm- related genes in WT and *Gas5* KO ESCs. Data normalized to Gapdh. (C) Proliferation curves of WT and *Gas5* KO ESCs measured by cell counting at 0, 12-, 24-, 36-, and 48-h post-seeding. (D) Cell cycle analysis of WT and *Gas5* KO ESCs. Histograms show the percentage of cells in G1, S, and G2/M phases. (E) Western blot of H3K9me3 in WT and *Gas5* KO ESCs. Bar plots show quantifications of H3K9me3 intensity normalized to α-Tubulin. (F) Immunofluorescence staining of H3K9me3 (green) in WT and *Gas5* KO ESCs. Nuclei were counterstained with DAPI (blue). Scale bars: 10 µm. The box plot shows the quantification of heterochromatin foci per cell. *n *= 165 (WT) and 165 (*Gas5* KO) cells pooled across three independent experiments. (G and H) Heat maps of SUV39H2 (G) and H3K9me3 (H) ChIP–seq signals in WT and *Gas5* KO ESCs. The scale below indicates the peak center (C) and 3 kb upstream/downstream of the peak. Color intensity represents fold change (IP/Input). (I) Representative genomic tracks of SUV39H2 and H3K9me3 ChIP–seq signals at the *Tfap2c* and *Stra8* gene loci in *Gas5* KO ESCs. The y-axis represents fold change (IP/Input). Data in (B–E) and are shown as mean ± SEM (*n *= 3 independent experiments).

Indeed, immunoblotting presented a substantial loss of H3K9me3 in *Gas5* KO ESCs (Fig. 4E). Immuno­fluorescence showed reduced H3K9me3 foci density and volume relative to empty vector (EV) controls (Fig. 4F). This H3K9me3 reduction was also observed in mouse embryonic fibroblasts (MEFs) under Gas5 interference (Fig. S4E). Concurrently, global H3K9me1 and H3K9me2 levels remained unchanged, while H3K27me3 and H3K14ac levels were significantly reduced (Fig. S4F). Western blot confirmed that the total protein abundance of SUV39H1, SUV39H2, and SETDB1 remained constant following *Gas5* knockout (Fig. S4G), and the protein level of major H3K9me3 demethylases (KDM4A/B/C/D) also remained unaltered (Fig. S4H and S4I). However, as revealed by ChIP-seq, SUV39H2 chromatin occupancy was largely abrogated, accordant with the global loss of H3K9me3, especially at the repetitive element regions (Figs. 4G–I and S4J). Consequently, this led to the aberrant derepression of these elements (Fig. S4L).

These data demonstrate that SUV39H2 recruitment to chromatin, rather than its protein stability or expression, is specifically dependent on the presence of lncRNA Gas5, thereby maintaining H3K9me3-marked heterochromatin.

### SUV39H2-Gas5 interaction is required for faithful mitosis and genome instability

To investigate how RNA modulates the chromatin occupancy and function of SUV39H2, we characterized the SUV39H2 interactome in the presence or absence of RNA. Using photo-crosslinking coupled with Flag-immunoprecipitation and mass spectrometry in ESCs expressing ^FB^SUV39H2, we identified protein complexes associated with SUV39H2 upon RNase A treatment or Gas5 depletion (Fig. 5A). GO analysis indicated that these RNA-dependent interactors were predominantly associated with nuclear processes, including chromatin remodeling, epigenetic regulation, RNA localization, and chromosome segregation (Fig. 5B). Specifically, LRWD1—an origin recognition complex (ORC)-associated protein regulating microtubule nucleation and cell cycle progression (Wang et al., 2017, 2018)—emerged as a prominent RNA-dependent interactor of SUV39H2 (Fig. 5A). To corroborate these finding, we performed RNA pulldown followed by mass spectrometry. Among the protein candidates, SUV39H2 and LRWD1 were found to interact with Gas5 RNA but not its antisense RNA (Fig. S5A). GO analysis revealed that Gas5-bound proteins are highly enriched in chromatin remodeling and DNA replication, exhibiting significant overlap with the SUV39H2 interactome (Fig. S5B). The interaction between SUV39H2 and LRWD1 was further validated by co-immunoprecipitation. Similar to the interaction with the known partner HP1α, the SUV39H2–LRWD1 association was severely compromised by RNase A treatment, Gas5 depletion, or loss of SUV39H2 RNA-binding activity (Figs. 5C, 5D and S5C).

**Figure 5.**
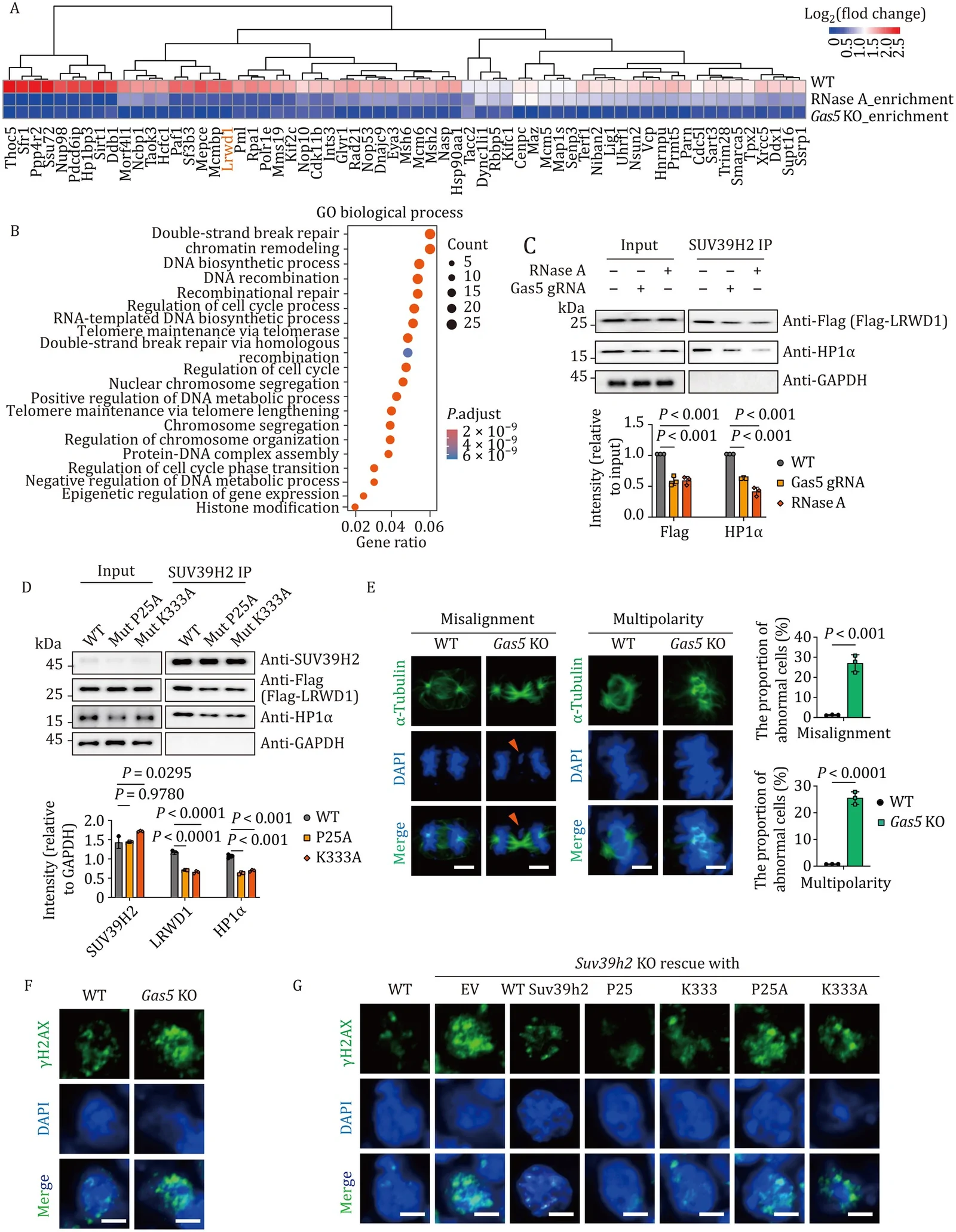
**SUV39H2-Gas5 interaction is required for faithful mitosis and genome instability**. (A) Heatmap of high-confidence SUV39H2-interacting proteins identified by immunoprecipitation mass spectrometry (IP-MS) in ESCs under three conditions: untreated, RNase A-treated, and *Gas5* KO. (B) GO analysis of RNA-dependent SUV39H2-interacting proteins identified by IP-MS in ESCs. (C) Co-immunoprecipitation (Co-IP) of endogenous SUV39H2 with LRWD1 and HP1α in ESCs under three conditions: untreated, RNase A-treated, and *Gas5* KO. Bar plots show quantifications of LRWD1 and HP1α intensity normalized to GAPDH. (D) Co-IP of endogenous SUV39H2 with LRWD1 and HP1α in WT and Mut (P25A/K333A) ESCs. Bar plots show quantifications of LRWD1 and HP1α intensity normalized to GAPDH. (E) Representative images of mitotic errors, including alignment defects and multipolar spindles. Bar plots show the percentage of abnormal cells. *n *= 110 (WT) and 110 (*Gas5* KO) cells pooled across three independent experiments. Scale bars: 5 μm. (F) Immunofluorescence staining of γH2AX foci (green) in WT and *Gas5* KO ESCs. Nuclei counterstained with DAPI (blue). Scale bars: 5 μm. (G) Immunofluorescence staining of γH2AX in *Suv39h2* KO ESCs rescued by WT SUV39H2, WT (P25/K333) or the Mut (P25A/K333A). Scale bar: 5 μm. Data in (C–E) are shown as mean ± SEM (*n *= 3 independent experiments).

To further investigate the cellular function of LRWD1, we depleted endogenous LRWD1 using small interfering RNAs (siRNAs). Similar to Gas5 depletion, knockdown of endogenous LRWD1 led to aberrant colony morphology in ESCs (Fig. S5D and S5E), together with impaired cell proliferation and S-phase arrest (Fig. S5F and S5G). Immunofluorescence showed a significantly higher frequency of chromosomal segregation errors in Gas5- or LRWD1-depleted mitotic cells, characterized by misaligned chromosomes, lagging chromosomes, and multipolar spindles (Figs. 5E and S5H). Furthermore, Gas5 deficiency also caused marked accumulation of double-stranded DNA breaks in ESCs (Figs. 5F and S5I). Importantly, *Suv39h2* KO ESCs recapitulated Gas5 depletion phenotypes, and could be effectively rescued by the overexpression of WT SUV39H2, but not by the RNA-binding-deficient mutants (Fig. 5G).

Collectively, these results indicate that the Gas5-dependent recruitment of SUV39H2 and its associated factors to heterochromatin is required for faithful chromosome segregation and the maintenance of genome stability.

## Discussion

In this study, we elucidated a critical RNA-mediated mechanism governing the recruitment and function of SUV39H2 in ESCs. We demonstrated that the chromatin association of SUV39H2 is strictly RNA-dependent. Loss of RNA interaction uncouples SUV39H2 from its genomic targets, leading to compromised H3K9me3-marked heterochromatin. Furthermore, we identified the lncRNA Gas5 as a specific RNA partner of SUV39H2, the elimination of which results in the loss of H3K9me3 and self-renewal ability of ESCs. Our data established that Gas5 acts as a mediator facilitating SUV39H2 function, thereby ensuring H3K9me3 maintenance and genomic stability.

The SUV39H family enzymes are the primary architects of constitutive heterochromatin. Although SUV39H1 and SUV39H2 share catalytic domains, SUV39H2 exhibits distinct expression patterns (Balan et al., 2021; O’Carroll et al., 2000). SUV39H2 harbors a unique N-terminal basic domain, which enables high-affinity binding to single-stranded RNAs, such as major satellite repeats, promoting the stable association of SUV39H2 with heterochromatin throughout mitosis (Camacho et al., 2017). Previous studies suggested that pericentromeric heterochromatin is not transcriptionally inert and the RNA transcripts may facilitate SUV39H recruitment (Camacho et al., 2017; Johnson et al., 2017). Our findings provide definitive evidence supporting RNA as an indispensable cofactor for SUV39H2. This adds a layer of regulatory plasticity, allowing rapid heterochromatin modulation via the turnover of specific RNAs.

Specifically, we identified lncRNA Gas5, which was originally discovered in a study exploring NIH 3T3 mouse fibroblast cells undergoing the G_0_ phase (quiescent cell state) (Coccia et al., 1992). Moreover, Gas5 was widely recognized as a potential tumor suppressor (Goustin et al., 2019) and has been well-characterized as a “sponge” for the glucocorticoid receptor (Hudson et al., 2014) or a regulator for self-renewal and pluripotency of ESCs (Tu et al., 2018). By demonstrating the requirement of Gas5 for SUV39H2’s function and H3K9me3 maintenance, we proposed a chromatin-architectural role for Gas5. Although the precise molecular mechanism remains to be fully elucidated, our preliminary data provide key insights. On one hand, RNase H treatment—which degrades RNA within RNA-DNA hybrids—significantly reduced SUV39H2 recruitment to genomic loci (data not shown), indicating that RNA-DNA hybrids are critical for SUV39H2 localization. On the other hand, RNA depletion and *Gas5* knockout altered the SUV39H2-binding protein profile, suggesting that RNA serves as a scaffold for SUV39H2 complex assembly. Further investigation is warranted to fully define the precise molecular and structural mechanisms underlying Gas5's functions. Consequently, *Gas5* KO-induced loss of H3K9me3 led to impaired chromatin-architectural, derepression of repetitive elements and genomic instability. This explains the severe cell cycle arrest and differentiation defects in Gas5-depleted ESCs, underscoring its contribution to self-renewal and genome surveillance.

Our findings reinforce the emerging paradigm that RNA is an integral component of chromatin-modifying complexes. For instance, the polycomb repressive complex 2 (PRC2), responsible for H3K27me3, binds RNAs with locus-specificity to regulate its activity (Pintacuda et al., 2017; Żylicz et al., 2019). Similarly, DNA methyltransferases (DNMTs) (Jeltsch and Jurkowska, 2016) and the LSD1 demethylase (Xu et al., 2024) have been shown to rely on RNA interactions for their catalytic function and localization. Our study adds SUV39H2 to this growing cohort of RNA-dependent epigenetic modifiers in mammals. The RNA dependence of epigenetic enzymes opens intriguing possibilities for site-specific regulation. While Gas5 functions as a primary and critical RNA partner for SUV39H2, it is highly plausible that other lncRNAs may play auxiliary, redundant, or complementary roles in directing SUV39H2 to specific heterochromatin. In the context of cell fate decision, the differential expression of specific lncRNAs could essentially “reprogram” the distribution of epigenetic modifications, thereby introducing to specific lineage commitment. Deciphering the RNA-guided targeting code that dictates the genomic binding sites of epigenetic modifiers will be crucial for understanding developmental programming and epigenetic disease pathogenesis.

## Supplementary Material

pwag039_Supplementary_Data

## Data Availability

Sequencing data generated in this study are available in the NCBI Gene Expression Omnibus (GEO) under accession number GSE327055.

## Code availability

No novel source code was presented in this study.

## Artificial intelligence (AI)

During the preparation of this work, AI tools (ChatGPT and DeepSeek) were used to check grammar and improve readability of the manuscript.

## Abbreviations

Act D: actinomycin D
ChIP: chromatin immunoprecipitation
Co-IP: co-immunoprecipitation
ESCs: embryo stem cells
GO: gene ontology
H3K9me3: H3 lysine 9 trim­ethylation
LncRNA: long non-coding RNA
RIP: RNA immunoprecipitation
