# Supplementary material for: LncRNA Gas5 directs SUV39H2 to establish heterochromatin and maintain genome stability
Source: Protein Cell. 2026 Jun 23;17(11):1035–45. doi: 10.1093/procel/pwag039 (PMC13721125; doi:10.1093/procel/pwag039)
Supplement: Supplementary file 1 [file pwag039_supplementary_data.pdf]

# Supplementary data for
# LncRNA Gas5 directs SUV39H2 to establish heterochromatin and maintain genome stability

Juntao Liu[1,4], Jiani Yang[1,4], Wen Ye[1], Dandan Zhang[1], Nan Su[1], Hong Wang[2], Yanping Zhang[2,3,*], Shaorong Gao[2,3,*] and Lan Kang[1,3,*]

[1] Institute for Regenerative Medicine, Shanghai East Hospital, Shanghai Institute of Stem Cell Research and Clinical Translation, Shanghai Key Laboratory of Signaling and Disease Research, Frontier Science Center for Stem Cell Research, School of Life Sciences and Technology, Tongji University, Shanghai 200092, China

[2] Clinical and Translation Research Center of Shanghai First Maternity & Infant Hospital, School of Life Sciences and Technology, Tongji University, Shanghai 200092, China

[3] Frontier Science Center for Stem Cell Research, Tongji University, Shanghai 200092, China

[4] These authors contributed equally: Juntao Liu, Jiani Yang.

[*] Corresponding authors:

Lan Kang, PhD

Tel: 86-21-65982276, Email: kanglan@tongji.edu.cn

Shaorong Gao, PhD

Tel: 86-21-65985182, Email: gaoshaorong@tongji.edu.cn

Yanping Zhang, PhD

Tel: 86-21-65982276, Email: yanpingzhang@tongji.edu.cn

Authors' ORCID: Lan Kang (0000-0003-4525-5923), Shaorong Gao (0000-0003-1041-3928), Yanping Zhang (0000-0003-3565-8120)

**This file includes:**

Materials and Methods, Supplementary Figures S1 to S5, Supplementary Tables S1.

# Supplementary Methods

## Cell Culture

Mouse embryonic stem cells (ESCs) on a feeder layer of mitomycin C-treated mouse embryonic fibroblasts. The cells were maintained in high-glucose Dulbecco's Modified Eagle's Medium (DMEM; Life Technologies, C11960500BT) supplemented with 15% fetal bovine serum (FBS; Gibco, 10099141), 2 mM L-Glutamine (Millipore Sigma, TMS-002-C), 0.1 mM non-essential amino acid (NEAA; Millipore, TMS-001-C), 1% of nucleosides mix (Millipore, ES-008-D), 0.1 mM β-mercaptoethanol (Gibco, 21985023) and 1000 U/mL recombinant leukemia inhibitory factor (LIF; Millipore, ESG1107). Cells were maintained at 37 °C in a humidified incubator with 5% $CO_2$. Cell lines were routinely tested for mycoplasma contamination.

## Embryoid Body (EB) Formation and Spontaneous Differentiation

To evaluate multi-lineage differentiation potential, WT, *Gas5* KO, and SUV39H2 mutant ESCs were subjected to an *in vitro* EB formation assay. Briefly, ESCs maintained in the pluripotent state were dissociated into single-cell suspensions and washed twice with PBS to completely remove Leukemia Inhibitory Factor. The cells were then resuspended in EB differentiation medium (ESCs culture medium without LIF). For EB generation, $2 \times 10^6$ cells were seeded into ultra-low attachment plates to promote 3D aggregation. The EBs were maintained in suspension culture at 37 °C under 5% $CO_2$, and the differentiation medium was carefully replaced every other day. After 3 days of spontaneous differentiation, the resulting EBs were collected by gravity sedimentation, washed with cold PBS, and pelleted for total RNA extraction and downstream RT-qPCR analysis of germ layer markers.

## Plasmids and short interfering RNA

Oligonucleotides encoding single guide RNAs (sgRNAs) targeting lncRNA Gas5 were cloned into a modified lentiCRISPR v2 vector, from which the Cas9 coding sequence had been deleted. To generate the inducible CRISPR system, HEK293T cells were co-transfected with the resulting sgRNA expression construct (or the Cas9-inducible expression plasmid pCW57.1), psPAX2, and pMD2.G packaging plasmids using VigoFect. Viral supernatants were collected 48 hours post-transfection and filtered through a 0.45-μm filter. Target cells were co-transduced with the sgRNA-carrying lentivirus and the Cas9-inducible lentivirus. After 48 hours, infected cells were selected with 1 μg/mL puromycin and 50 μg/mL hygromycin for 7 days to establish stable cell lines. To induce Cas9 expression, cells were treated with 1 μg/mL doxycycline for 12 hours.

For short interfering RNA (siRNA) experiments, cells were transfected with siRNA targeting lncRNA Gas5 or a non-targeting scramble control using Lipofectamine 3000 transfection reagent (Thermo Scientific, L3000008), according to the manufacturer's instructions.

**Chromatin Immunoprecipitation and Sequencing (ChIP-seq)**

Cells were cultured in 15-cm dishes until reaching 70–80% confluency. The medium was removed, and cells were washed once with phosphate-buffered saline (PBS). Cross-linking was performed by incubating cells with 10 mL of 1% (v/v) formaldehyde in PBS for 10 minutes at room temperature, followed by quenching with glycine at a final concentration of 125 mM. Subsequently, cells were scraped, collected, and washed twice with cold PBS containing 0.5% Igepal CA-630 (Sigma-Aldrich, I8896) to isolate nuclei.

For each sample, cell pellets corresponding to $1 \times 10^7$ nuclei were resuspended in 500 μL of ChIP lysis buffer (50 mM Tris-HCl pH 8.0, 10 mM EDTA, 0.5% SDS, 1× Protease inhibitor solution (Roche, 04693132001)). The lysate was incubated on ice for 10 minutes and then sonicated using a Covaris S220 focused-ultrasonicator to achieve

a DNA fragment size range of 200–300 bp. The sonicated lysates were centrifuged at 14,000 × *g* for 10 minutes at 4 °C to remove insoluble debris. To reduce non-specific binding, the soluble chromatin supernatant was pre-cleared by incubation with 20 μL of Pierce Protein A/G Magnetic Beads in IP dilution buffer (16.7 mM Tris-HCl pH 8.0, 1.2 mM EDTA, 167 mM NaCl, 1% Triton X-100) for 1 hour at 4 °C. An aliquot of the pre-cleared chromatin was reserved as the "Input" control.

For immunoprecipitation, 200 μL of pre-cleared chromatin was incubated with 2 μL of anti-H3K9me3 antibody (Active Motif, 39161) or 2 μL of anti-SUV39H2 antibody (Abcam, ab190870) in IP dilution buffer overnight at 4 °C with rotation. Subsequently, 10 μL of Pierce Protein A/G magnetic beads were added to each IP reaction and incubated for 2 hours at room temperature with rotation. The beads were captured using a magnetic rack and washed sequentially (twice per buffer, 1 mL each) with low salt wash wash buffer (20 mM Tris-HCl pH 8.0, 2 mM EDTA, 150 mM NaCl, 0.1% SDS, 1% Triton X-100), high salt wash buffer (20 mM Tris-HCl pH 8.0, 2 mM EDTA, 500 mM NaCl, 0.1% SDS, 1% Triton X-100), LiCl wash buffer (10 mM Tris-HCl pH 8.0, 1 mM EDTA, 250 mM LiCl, 1% sodium deoxycholate, 1% NP-40 substitute) and TE buffer (10 mM Tris-HCl pH 8.0, 1 mM EDTA).

Immunoprecipitated chromatin was eluted by incubation with 120 μL of elution buffer (100 mM $NaHCO_3$, 1% SDS) for 30 minutes at room temperature with rotation. The reserved input control was processed simultaneously by adding an equivalent volume of elution buffer. Cross-links were reversed by adding NaCl to a final concentration of 200 mM and incubating overnight at 65 °C. Samples were then digested with proteinase K (New England Biolabs, P8107S) in the presence of 1 M Tris-HCl (pH 6.5) and 500 mM EDTA for 1 hour at 55 °C. DNA was purified by phenol-chloroform-isoamyl alcohol (25:24:1) extraction followed by ethanol precipitation with glycogen (Roche, 10901393001) as a carrier. Purified DNA was resuspended in 25 μL of 10 mM Tris-HCl (pH 8.0) and quantified using a Qubit fluorometer (Thermo Fisher Scientific).

Sequencing libraries were prepared from 25 ng of ChIP DNA using the KAPA HyperPlus Kit (Roche, KK8512) following the manufacturer's instructions. Libraries were pooled equimolarly and sequenced on an Illumina NextSeq 550 using a High Output v2.5 kit (150 cycles, paired-end).

**RNase A Treatment**

RNA dependent ChIP-seq experiments for SUV39H2 and RPB1 were performed with RNase A treatment. Specifically, 5 μg/mL of DNase- and protease-free RNase A (Thermo Scientific, EN0531) was added directly to the immunoprecipitation reaction (concurrently with the primary antibody) for wild-type ESCs. All subsequent steps, including washing, elution, and DNA library preparation, were performed as described in the 'ChIP-seq' section above.

**Immunofluorescence Staining**

Cells were fixed with 4% paraformaldehyde (PFA) for 10 minutes at room temperature, permeabilized with 0.1% Triton X-100 in PBS for 10 minutes, and blocked with 5% donkey serum for 1 hour at room temperature. Immunofluorescence staining was performed to detect SUV39H2 and H3K9me3. First, cells were incubated overnight at 4 °C with anti-SUV39H2 antibody (1:200; Abcam, ab190870) diluted in 0.5% donkey serum. After washing three times with PBS, cells were incubated for 1 hour at room temperature with Alexa Fluor 488-conjugated donkey anti-rabbit IgG (1:500; Invitrogen, A21206). To preventing cross-reactivity between the sequential staining steps, cells were incubated with 5% rabbit serum for 1 hour at room temperature to saturate open binding sites on the first secondary antibody. Following this blocking step, cells were incubated overnight at 4 °C with the second primary antibody, anti-H3K9me3 (1:200; Active Motif, 39161). Cells were then washed three times with PBS and incubated for 1 hour at room temperature with Alexa Fluor 647-conjugated donkey anti-rabbit IgG (1:500; Invitrogen, A10277). Finally, nuclei were counterstained with

1 μg/mL DAPI (Invitrogen, D3571) for 5 minutes, and coverslips were mounted on glass slides using an anti-fade mounting medium.

**Western Blotting**

Cells were lysed in RIPA buffer supplemented with protease and phosphatase inhibitors. Protein samples were mixed with 2× Laemmli buffer (1:1 ratio) and denatured at 95 °C for 8 minutes. Proteins were separated by SDS-PAGE and electrophoretically transferred onto polyvinylidene difluoride (PVDF) membranes. Membranes were blocked with 5% bovine serum albumin (BSA) in PBS containing 0.1% Tween-20 (PBS-T) for 1 hour at room temperature. Subsequently, membranes were incubated overnight at 4 °C with primary antibodies diluted in 0.5% BSA/PBS-T. The primary antibodies used were anti-H3K9me3 (1:1000; Active Motif, 39161), anti-SUV39H2 (1:1000; Abcam, ab190870), anti-α-Tubulin (1:5000; Proteintech, 66031-2-ig), anti-GAPDH (1:5000; Proteintech, 60004-1-Ig), anti-H3K9me1 (1:1000; Thermo Fisher Scientific, MA5-33385), anti-H3K9me2 (1:1000; Abcam, ab1220), anti-H3K4me3 (1:1000; Diagenode, C15410030), anti-H3K27me3 (1:1000; Diagenode, C15410195) and anti-H3 (1:1000; Cell Signaling Technology, 4499S).

Following primary incubation, membranes were washed with PBS-T and incubated with horseradish peroxidase (HRP)-conjugated secondary antibodies (anti-mouse or anti-rabbit IgG; 1:5000) for 1 hour at room temperature. Protein bands were visualized using SuperSignal™ West Pico PLUS Chemiluminescent Substrate (Thermo Scientific, 34580) and detected using a chemiluminescence imaging system.

**RNA Immunoprecipitation (RIP) and Sequencing**

RIP experiments were performed using the EZ-Magna RIP Kit (Millipore, 17-10470) with minor modifications. Briefly, ESCs were cultured in 15-cm dishes until reaching 70–80% confluency. The culture medium was removed, and cells were washed once with PBS. To isolate nuclei, cells were collected and washed twice with cold PBS

containing 0.5% Igepal CA-630. For each sample, cell pellets corresponding to $2 \times 10^7$ cells were resuspended in 100 μL of RIP Lysis Buffer supplemented with 0.5 μL Protease Inhibitor Cocktail and 0.25 μL RNase Inhibitor. The suspension was incubated at -80 °C for 20 minutes, thawed on ice, and incubated on ice for an additional 20 minutes to ensure complete lysis. Lysates were diluted with 900 μL of RIP Immunoprecipitation Buffer (comprising 860 μL RIP Wash Buffer, 17.5 mM EDTA pH 8.0, 20 μL Protease Inhibitor Cocktail, and 5 μL RNase Inhibitor). Insoluble debris was removed by centrifugation at 13,000 × *g* for 10 minutes at 4 °C. An aliquot of the supernatant was saved as the "Input" control.

Immunoprecipitation was performed by incubating the remaining lysate with 2 μg of anti-SUV39H2 antibody (Abcam, ab190870) or normal rabbit IgG (as a negative control) and 10 μL Protein A/G Magnetic Beads for 4 hours at 4 °C with rotation. Following incubation, the beads were washed six times with cold RIP Wash Buffer. To release the RNA, the protein-RNA complexes were digested with Proteinase K for 30 minutes at 55 °C. Co-precipitated RNA was then extracted using TRIzol Reagent (Thermo Scientific, 15596026) and treated with TURBO DNase (Thermo Scientific, AM2238) to remove genomic DNA. Purified RNA was used for library preparation using the KAPA Stranded RNA-Seq Kit (Roche, KK8483) according to the manufacturer's instructions, which includes RNA fragmentation to 200-300 bp. Libraries were pooled equimolarly and sequenced on an Illumina HiSeq 4000 system using a HiSeq 4000 High Output PE150 Cluster kit.

**RNA-seq**

Libraries were prepared from 100 ng of total RNA using the KAPA Stranded RNA-Seq Kit with RiboErase (HMR) (Roche, KK8483), which incorporates a ribosomal RNA depletion step, following the manufacturer's instructions. Library concentration and quality were assessed prior to pooling. Sequencing was performed on an Illumina HiSeq 4000 system to generate 150-bp paired-end reads.

## Quantitative Real–Time PCR (qPCR)

Total RNA was extracted using TRIzol Reagent (Thermo Scientific, 15596026). Complementary DNA (cDNA) was synthesized from 1 μg total RNA using the 5× All-In-One RT MasterMix (ABM, G490) according to the manufacturer's instructions. Quantitative PCR (qPCR) was performed using SYBR Premix Ex Taq II (Tli RNaseH Plus) (Takara Bio, RR820A) on an ABI 7500 Fast Real-Time PCR System. All reactions were performed in biological and technical triplicates. Relative gene expression levels were normalized to the internal control Gapdh and quantified using the comparative $2^{-\Delta\Delta Ct}$ method. Primer sequences for all genes are listed in **Supplementary Table 1**.

## Cytosolic/Nuclear Fractionation

Subcellular fractionation was performed using a differential detergent lysis protocol. Cells were harvested and incubated in ice-cold hypotonic buffer (25 mM Tris-HCl pH 7.4, 1 mM $MgCl_2$, 5 mM KCl) for 5 minutes on ice. Subsequently, an equal volume of ice-cold hypotonic buffer containing 0.5% NP-40 substitute was added, and samples were incubated on ice for an additional 5 minutes to disrupt cell membranes. The lysate was centrifuged at 5,000 × *g* for 5 minutes at 4 °C. The resulting supernatant (cytosolic fraction) was collected. The nuclear pellet was washed once with hypotonic buffer and then was resuspended in nuclear extraction buffer (20 mM HEPES pH 7.9, 400 mM NaCl, 1 mM EDTA, 1 mM EGTA, 1 mM dithiothreitol (DTT), 1 mM phenylmethylsulfonyl fluoride). The nuclear suspension was incubated at 4 °C for 30 minutes with vigorous vortexing every 10 minutes. Finally, insoluble debris was removed by centrifugation at 12,000 × *g* for 10 minutes at 4 °C, and the supernatant was collected as the nuclear extract.

## In Vitro Transcription and RNA 3′-End Biotinylation

Prior to in vitro transcription, plasmids pcDNA3.1-T7-Gas5 and pcDNA3.1-T7-Gas5-Antisense were linearized using the restriction enzyme SmaI (NEB, R0141L). Primer sequences are listed in **Supplementary Table 1**. Linearized DNA templates were purified and quantified. In vitro transcription was performed using the mMESSAGE mMACHINE™ T7 ULTRA Transcription Kit (Thermo Fisher Scientific, AM1345) according to the manufacturer's protocol. Following transcription, the template DNA was removed by digested with TURBO DNase (Thermo Fisher Scientific, AM2239) for 15 minutes at 37 °C. The resulting RNA transcripts were purified using the RNeasy Mini Kit (Qiagen, 74104) and quantified.

For 3′-end labeling, purified RNA was incubated with the Pierce™ RNA 3' End Biotinylation Kit (Thermo Fisher Scientific, 20160) following the manufacturer's instructions to attach a biotin tag. The labeled RNA was then purified using TRIzol Reagent followed by ethanol precipitation. RNA concentration was measured using a NanoDrop spectrophotometer (Thermo Fisher Scientific), and RNA integrity was assessed by denaturing agarose gel electrophoresis.

**RNA Pull-down Assay**

The RNA pull-down assay was performed as described below. To facilitate proper RNA secondary structure formation, 2 μg of 3′-end biotinylated Gas5 or Gas5-Antisense control RNA was heated at 65 °C for 10 minutes in RNA folding buffer (10 mM Tris-HCl pH 7.0, 100 mM KCl, 10 mM $MgCl_2$) and then slowly cooled to room temperature for 20 minutes.

Nuclear extracts were prepared from target cells using the Magna Nuclear RIP Kit (Millipore, 17-10520), omitting the sonication step to preserve protein integrity. The folded bait RNA was incubated with 500 μg of nuclear extract overnight at 4 °C with gentle rotation. To stabilize transient interactions, RNA-protein complexes were crosslinked using 254 nm UV light (400 $mJ/cm^2$). Dynabeads™ MyOne™ Streptavidin C1 beads (Thermo Fisher Scientific, 65002) were pre-washed and equilibrated in RIP

wash buffer (supplied with kit). The crosslinked complexes were then incubated with 50 μL of the bead slurry for 2 hours at 4 °C. Beads were captured magnetically and washed five times (1 mL per wash) with RIP wash buffer. Bound proteins were eluted by boiling beads in 2× Laemmli sample buffer for 10 minutes at 95 °C. Eluted proteins were separated by SDS-PAGE for subsequent mass spectrometry or western blot analysis.

**RNA Fluorescence in Situ Hybridization (RNA-FISH) and Immunofluorescence**

To detect the subcellular localization of lncRNA Gas5, RNA-FISH was performed using in vitro-transcribed, biotinylated antisense probes. Cells cultured on coverslips were fixed in 3.6% PFA containing 10% acetic acid for 15 minutes at room temperature. Following fixation, cells were permeabilized with 0.5% Triton X-100 in PBS for 5 minutes on ice. Hybridization was performed by incubating cells overnight at 50 °C with 100 ng/mL denatured biotinylated probes in hybridization buffer (50% formamide, 2× SSC, 0.1% Tween-20, 2 mM vanadyl ribonucleoside complex). Post-hybridization, cells were washed sequentially with 2× SSC/50% formamide (3 × 5 minutes at 50°C), 1× SSC (2 × 5 minutes at room temperature), and PBS (2 × 5 minutes at room temperature). For signal detection, samples were blocked with 5% donkey serum for 1 hour at room temperature and then incubated with a goat anti-biotin antibody (1:50; Thermo Fisher, 31852) overnight at 4°C. After washing with PBS-T, samples were incubated with Alexa Fluor 488 donkey anti-goat IgG (1:500; Thermo Fisher, A-21202) for 1 hour at room temperature. Nuclei were counterstained with 1 μg/mL DAPI for 5 minutes. Coverslips were mounted using ProLong™ Diamond Antifade Mountant. Images were acquired using a ZEISS LSM 880 confocal microscope with a 63× oil objective.

**Cell Cycle Analysis**

Cells were harvested and fixed in ice-cold 70% ethanol for at least 1 hour at 4 °C. Fixed cells were pelleted by centrifugation at 1,000 × *g* for 5 minutes. The pellets were resuspended in PBS containing 0.25% Triton X-100 and incubated on ice for 15 minutes to ensure permeabilization. After centrifugation, cells were resuspended in 0.5 mL of PBS staining solution containing 10 μg/mL RNase A (Thermo Fisher, EN0531) and 20 μg/mL propidium iodide (Sigma-Aldrich, P4170). The suspension was incubated in the dark at room temperature for 30 minutes. Cell cycle distribution was analyzed using a BD Accuri™ C6 flow cytometer (BD Biosciences). At least 10,000 events were recorded per sample. The data were analyzed using FlowJo software (v10.8.1, BD Biosciences), and the percentage of cells in G0/G1, S, and G2/M phases was determined using the Dean-Jett-Fox model.

**Co-Immunoprecipitation (Co-IP)**

ESCs were harvested and lysed on ice for 30 minutes in IP lysis buffer (50 mM Tris-HCl pH 7.5, 150 mM NaCl, 0.5% NP-40 substitute, 1 mM PMSF, 1× protease inhibitor cocktail, 1 U/μL RNase inhibitor). To ensure nuclear protein extraction, lysates were sonicated with three 10-second pulses (30% amplitude, 0.5 sec on/off cycle) on ice. The lysates were then clarified by centrifugation at 16,000 × *g* for 15 minutes at 4 °C. An aliquot of the supernatant was reserved as the "Input" control.

For immunoprecipitation, 500 μg of total protein was first pre-cleared by incubation with 10 μL of Protein A/G Magnetic Beads for 1 hour at 4 °C to reduce non-specific binding. The supernatant was collected and incubated overnight at 4 °C with rotation in the presence of 2 μg of anti-SUV39H2 antibody and 20 μL Protein A/G Magnetic Beads. Following incubation, the beads were captured and washed four times with 1 mL of high salt wash buffer (50 mM Tris-HCl pH 7.5, 300 mM NaCl, 0.05% sodium deoxycholate, 0.5% NP-40 substitute, 1 mM PMSF). Bound protein complexes were eluted by resuspending the beads in 40 μL of 2× Laemmli buffer and boiling at

95 °C for 8 minutes. The eluates and the reserved Input control were analyzed by SDS-PAGE and immunoblotting.

## Quantification and statistical analysis

### ChIP-seq Data Analysis

Sequencing reads were trimmed to remove adapters using Trim Galore (v0.6.7) with the parameter--paired. Trimmed reads were aligned to the mouse reference genome (mm9) using Bowtie2 (v2.4.4) (Langmead and Salzberg, 2012) with options--no-mixed and--no-discordant. Aligned reads were filtered to retain uniquely mapped reads using SAMtools (v1.3) (Li et al., 2009) view-F 4, and duplicate reads were removed with Picard MarkDuplicates (v2.23.8). Filtered BAM files were indexed using SAMtools and converted to normalized BigWig files (RPKM) using bamCoverage (v3.5.5) from deepTools for visualization. The normalized BigWig files of ChIP and input samples were used to quantify the averaged signal at designated regions by bigWigSummary (Kent et al., 2010), and the ratio (IP/input) was regarded as ChIP signal intensity. Signal tracks and peak distributions were generated using deepTools and IGV (Thorvaldsdóttir et al., 2013).Peaks were called using MACS2 (v2.1.1)(Zhang et al., 2008) on pooled replicates with parameters-g mm--nolambda--nomodel--keep-dup all, and annotated against the mm9 genome using ChIPseeker (v1.38.0)(Yu et al., 2015). Peaks overlapping ENCODE blacklist (Amemiya et al., 2019) regions were excluded. Signal profiles around transcription start sites (TSS ± 3 Kb) were generated using deepTools.

The sequencing reads were counted in a strand-specific way for gene bodies by using featureCounts (v2.0.6)(Liao et al., 2014). Read counts were normalized using DESeq2 (v1.26.0)(Love et al., 2014), and fold changes with adjusted *p*-values ($\alpha = 0.05$) were calculated via fdrtool (v1.2.15)(Strimmer, 2008). Significant genes in comparisons were visualized using ggplot2 (v3.3.3) scatter plots.

To compare the enrichment of ChIP peaks on different elements, genomic regions of promoter (TSS ± 2 Kb, 2Kb upstream of the transcription start site), exons, introns

and retrotransposons were defined; the percentage of peaks falling on each element was then calculated, which is further divided by the percentage of input reads falling on the corresponding element, and this ratio is regarded as the relative enrichment for the element.

### RIP-seq Data Analysis

Paired-end reads were trimmed using Trim Galore (v0.6.7) and aligned to mm9 using STAR (v2.7.11)(Dobin et al., 2013). Duplicate reads were removed with Picard MarkDuplicates (v2.23.8). Peaks were called using MACS2 (v2.1.1) (Zhang et al., 2008) with parameters-g mm --nolambda --nomodel --keep-dup all, and high-confidence peaks were defined as overlaps between replicates using BEDTools (v2.30.0)(Quinlan and Hall, 2010). Peaks were annotated to genomic features (promoters, exons, introns, 3' UTR, 5' UTR) using ChIPseeker (v1.38.0)(Yu et al., 2015) and summarized for relative enrichment by dividing the percentage of peaks on each element by the percentage of input reads on corresponding regions. Gene ontology (GO) analysis of peak-associated genes was performed using clusterProfiler (v4.0.5) for biological processes (BP) at a significance threshold of $p < 0.05$. Signal tracks and peak distributions were generated using deepTools and IGV(Thorvaldsdóttir et al., 2013).

### RNA-seq Data Analysis

The sequencing reads were first trimmed using Trim Galore (v0.6.7) with the --paired option to remove adapter sequences. The trimmed reads were aligned to the mouse reference genome (mm9) using STAR (v2.7.11)(Dobin et al., 2013) with the parameters --runThreadN 20 and --outSAMtype BAM SortedByCoordinate. Post-alignment, the uniquely mapped reads were assessed using log files generated by STAR, and key mapping statistics were extracted, including the number of input reads, uniquely mapped reads, and mapping percentage. Normalized coverage files were generated

using bamCoverage (v3.5.5) with RPKM normalization, and BAM files were indexed using samtools index.

Gene expression quantification was performed using Cufflinks (v2.2.1)(Trapnell et al., 2010), which estimated FPKM values based on the aligned reads. To ensure the uniqueness of genes, the FPKM data was filtered and processed, resulting in a list of unique FPKM values for each sample. For a more detailed count-based analysis, featureCounts (v2.0.6)(Liao et al., 2014) was used to quantify the number of reads mapping to exons, using the mm9 gene annotation (GTF format), and then differentially expressed genes (DEGs) were identified by DESeq2(Love et al., 2014) with adjusted P value <0.05 and requiring at least 2 reads in at least one samples.

**Statistics and reproducibility**

Statistical analyses were performed in R v.4.3.1. At least two biological replicates were used in each experiment unless otherwise stated. p values less than 0.05 were considered to indicate statistical significance. Unpaired two-sided t-tests were used to calculate the p values indicated in the figure legends, for assessment of the statistical significance of differences between groups. In all statistical comparisons involved in sequencing experiments, p values were corrected for multiple testing by false discovery rate (FDR).

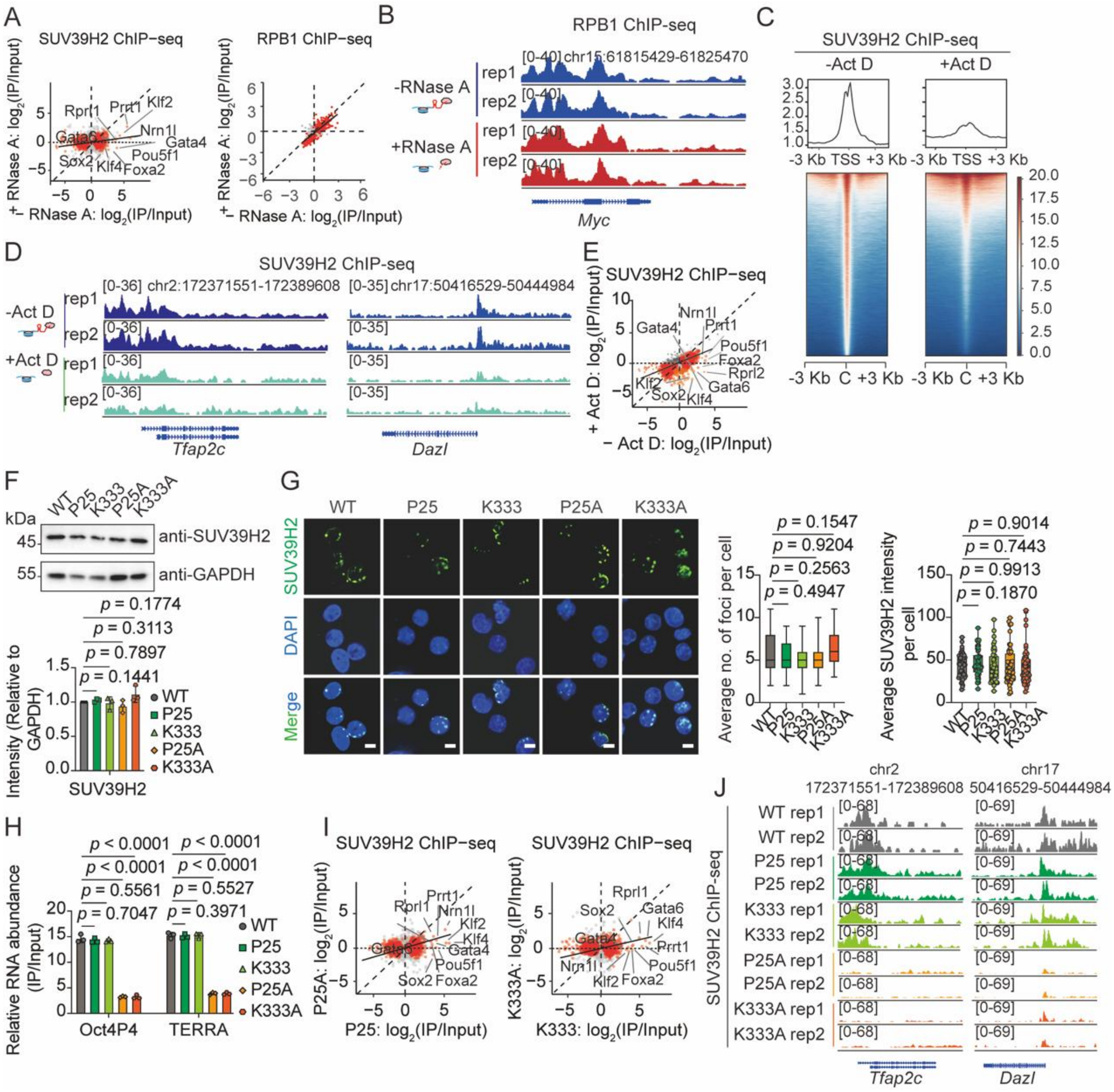


**Supplementary Figure 1. Interaction with RNA is required for SUV39H2 recruitment to chromatin.**

(A) Gene scatter plot of SUV39H2 and RPB1 rChIP–seq. RNase A treatment (y-axis) was compared to control (x-axis) in terms of fold change (log scale) of genes in IP relative to Input. Three independent clones in each condition were used. Ten enriched genes in SUV39H2 ChIP-seq (−RNase A) are labeled.

(B) Representative genomic tracks of RPB1 rChIP–seq experiments. The *Myc* loci is shown in RPB1 rChIP–seq data. The y axis shows fold change (IP/Input), with −RNase A (blue) and +RNase (red).

(C) Heat maps of SUV39H2 ChIP in control (-Act D) and Act D-treated (+Act D) ESCs. The scale below indicates the peak center (C) and the 3 kilobases (3 Kb) upstream/downstream of the peak. Color intensity represents fold change (IP/Input).

(D) Representative genomic tracks of rChIP–seq experiments. The *Tfap2c* and *Dazl* loci are shown in SUV39H2 ChIP–seq data. The y axis shows fold change (IP/Input). −Act D (blue) and +Act D (green).

(E) Gene scatter plot of the SUV39H2 ChIP–seq in -Act D and +Act D ESCs. Act D treatment (y-axis) was compared to control (x-axis) in terms of fold change (log scale) of genes in IP relative to Input. Three independent clones in each condition were used. Ten enriched genes in SUV39H2 ChIP-seq (−Act D) are labeled.

(F) Western blot of SUV39H2 in WT, WT (P25/K333) and Mut (P25A/K333A) ESCs. Bar plots show quantifications of SUV39H2 intensity normalized to GAPDH.

(G) Immunofluorescence staining of SUV39H2 (green) in WT, WT (P25/K333) and Mut (P25A/K333A) ESCs. Nuclei counterstained with DAPI (blue). Scale bar: 10 µm. Bar plots shows the quantification of SUV39H2 foci and average intensity per cell. n = 65 cells pooled across three independent experiments.

(H) RIP-qPCR analysis of SUV39H2 binding to lncRNA Oct4P4 and TERRA in WT, WT (P25/K333) and Mut (P25A/K333A) ESCs.

(I) Gene scatter plot of SUV39H2 ChIP–seq in WT, WT (P25/K333) and Mut (P25A/K333A) ESCs. P25A/K333A treatment (y-axis) was compared to P25/K333 (x-axis) respectively in terms of fold change (log scale) of genes in IP relative to Input. Three independent clones in each condition were used. Ten enriched genes in SUV39H2 ChIP-seq are labeled.

(J) Representative genomic tracks of SUV39H2 ChIP–seq experiments. The *Tfap2c* and *Dazl* gene loci are shown in WT, WT (P25/K333) and Mut (P25A/K333A). The y axis shows fold change (IP/Input), with WT, WT (P25/K333) and Mut (P25A/K333A).

Data in F and H are shown as mean ± SEM. (n=3 independent experiments).

**Alt Text:** A ten-panel supplementary figure (A-J) supporting the requirement of RNA for SUV39H2 chromatin recruitment. Panels A, E, and I are gene scatter plots comparing fold changes under various treatments and genetic mutations. Panels B, D, and J display representative genomic signal tracks showing sequencing peaks across specific gene loci. Panel C features heat maps of ChIP signals aligned to genomic peak centers. Panel F displays Western blot bands alongside a bar chart for protein quantification. Panel G pairs fluorescence microscopy images with a bar chart quantifying cellular foci. Panel H is a bar chart presenting qPCR analysis results.

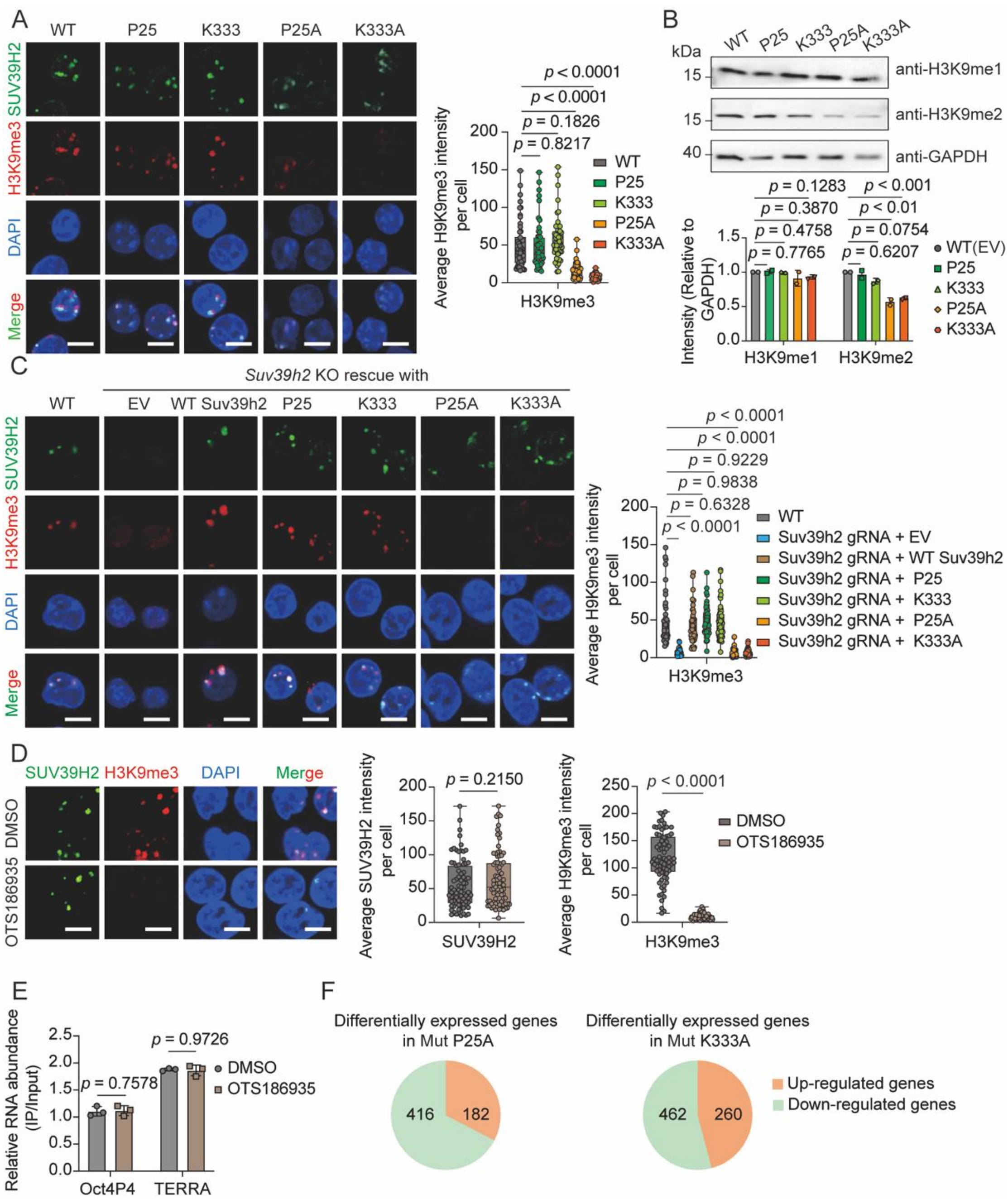


**Supplementary Figure 2. RNA binding is indispensable for SUV39H2-mediated heterochromatin maintenance.**

(A) Immunofluorescence stanning of SUV39H2 (green) and H3K9me3 (red) in WT, WT (P25/K333) and Mut (P25A/K333A) ESCs. Nuclei counterstained with DAPI (blue). Representative images from at least three independent experiments are presented. Scale bar: 10 μm. The box plot shows the quantification of heterochromatin foci per cell. n = 65 cells pooled across three independent experiments.

(B) Western blot of H3K9me1 and H3K9me2 in WT, WT (P25/K333) and Mut (P25A/K333A) ESCs. Bar plots show quantifications of H3K9me1 and H3K9me2 intensity normalized to GAPDH.

(C) Immunofluorescence stanning of SUV39H2 (green) and H3K9me3 (red) in *Suv39h2* KO mouse ESCs recued with WT SUV39H2, WT (P25/K333) and Mut (P25A/K333A). Nuclei counterstained with DAPI (blue). Scale bar: 10 μm. The box plot show quantifications of heterochromatin foci per cell. n = 65 cells pooled across three independent experiments.

(D) Immunofluorescence stanning of SUV39H2 (green) and H3K9me3 (red) in ESCs treated with DMSO and OTS186935. Nuclei counterstained with DAPI (blue). Scale bar: 10 μm. The box plot show quantifications of SUV39H2 and H3K9me3 foci per cell. n = 85 cells pooled across three independent experiments.

(E) RIP-qPCR analysis of SUV39H2 binding to lncRNA Oct4P4 and TERRA in ESCs treated with DMSO and OTS186935.

(F) Pie charts represent the number of differentially expressed genes in the indicated Mut (P25A/K333A), where differential expression analysis was carried out with respect to the WT (P25/K333). The group represents all the differentially expressed genes for a given mutant line. Numbers within the charts indicate the count of genes that are up (red) or down (green) regulated.

Data in B and E are shown as mean ± SEM. (n=3 independent experiments).

**Alt Text:** A six-panel supplementary figure (A-F) demonstrating that RNA binding is indispensable for SUV39H2-mediated heterochromatin maintenance. Panels A, C, and D pair fluorescence microscopy images with box plots to quantify cellular foci under various genetic and treatment conditions. Panel B displays Western blot bands alongside bar charts for protein quantification. Panel E is a bar chart presenting RIP-qPCR analysis results. Panel F features pie charts detailing the count of differentially expressed genes.

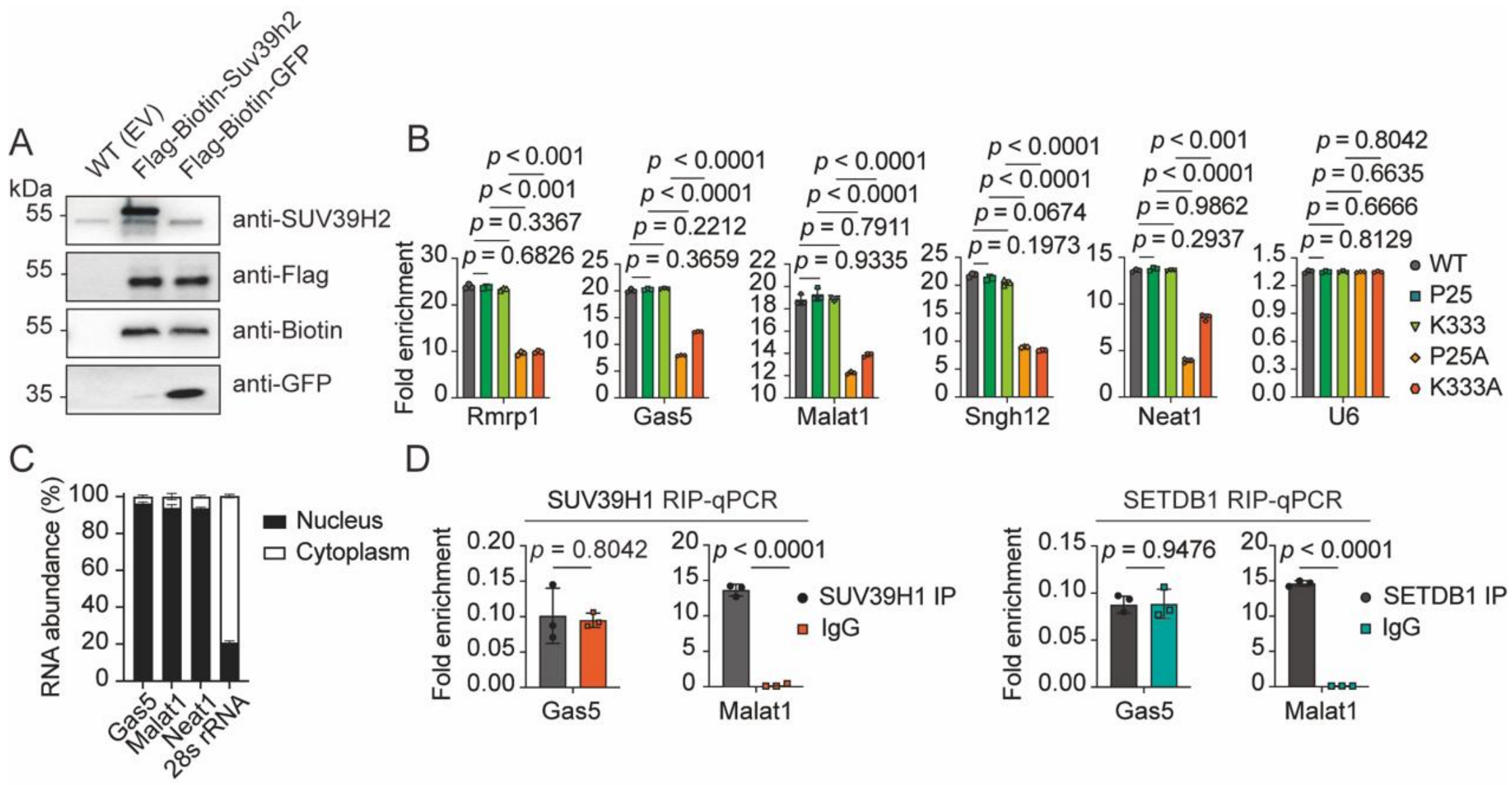


**Supplementary Figure 3. SUV39H2 specifically associates with the nuclear lncRNA Gas5.**

(A) Western blot of SUV39H2, Flag and GFP in Flag-Biotin-SUV39H2, Flag-Biotin-GFP versus WT ESCs.

(B) RIP-qPCR analysis of SUV39H2 binding to Rmrp, Malat1, Gas5, Snhg12, and Neat1 in ESCs. U6 RNA was used as controls.

(C) qPCR analysis of Gas5, Malat1, Neat1, and 28S rRNA levels in cytoplasmic and nuclear fractions of ESCs. The nucleus-enriched Neat1 and cytoplasm-enriched 28s rRNA were used as controls.

(D) RIP-qPCR analysis of SUV39H2 and SETDB1 binding to Gas5 and Malat1 in ESCs.

Data in B-D are shown as mean ± SEM. (n=3 independent experiments).

**Alt Text:** A four-panel supplementary figure (A-D) demonstrating that SUV39H2 specifically associates with the nuclear lncRNA Gas5. Panel A displays Western blot bands comparing protein expression across different engineered cell lines. Panels B, C, and D feature bar charts presenting quantitative PCR (qPCR) and RIP-qPCR analysis results across various RNA transcripts and subcellular fractions.

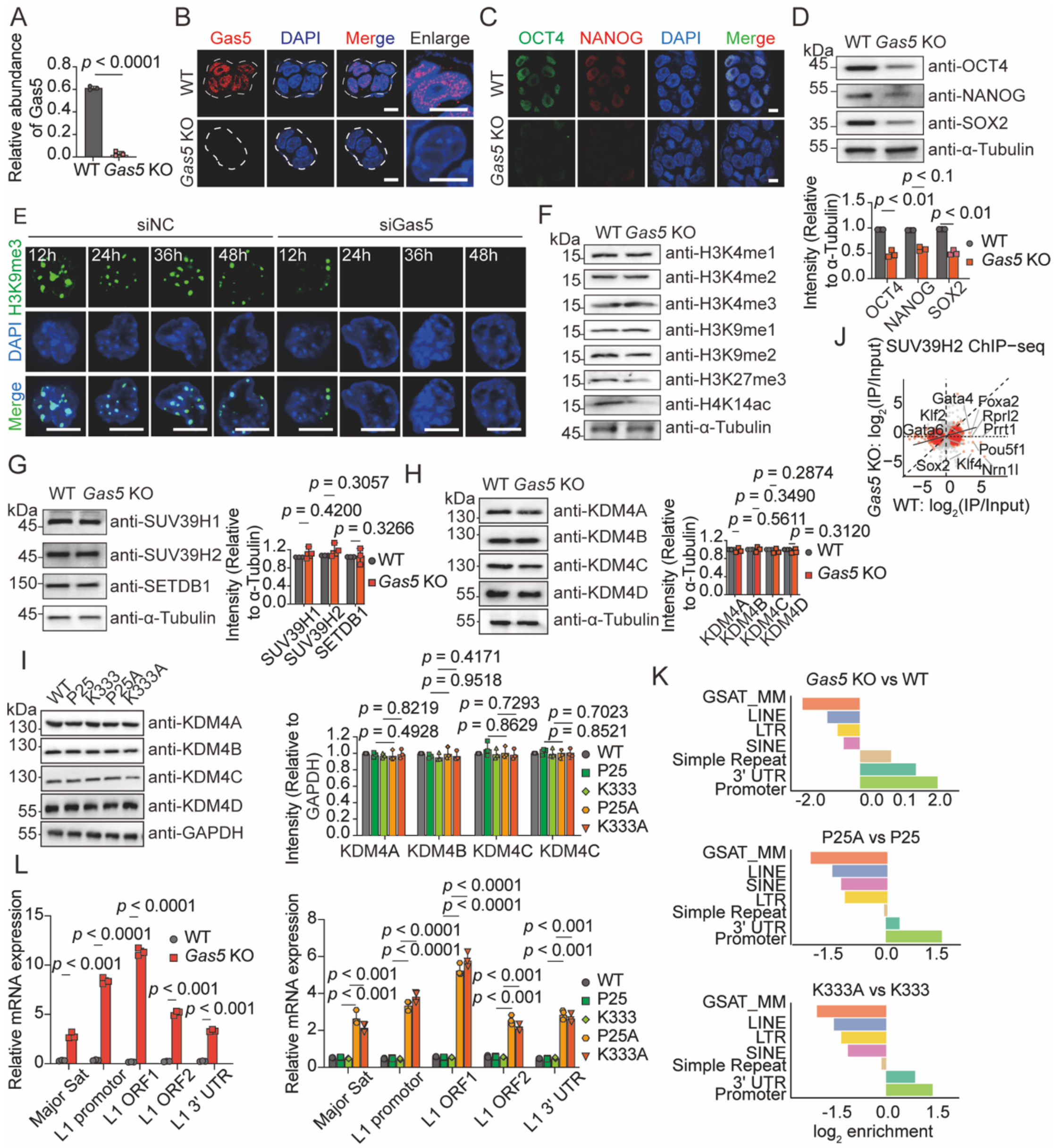


**Supplementary Figure 4. LncRNA Gas5 maintains heterochromatin integrity and SUV39H2 occupancy.**

(A) qPCR analysis of Gas5 in WT and *Gas5* KO ESCs. Data normalized to Gapdh.

(B) RNA FISH staining of Gas5 (red) in WT and *Gas5* KO ESCs. Nuclei were counterstained with DAPI (blue). Representative images from three independent clones are presented. Scale bars, 10 μm.

(C) Immunofluorescence staining of pluripotency-related protein OCT4 (green) and NANOG (red) in WT and *Gas5* KO ESCs. Nuclei counterstained with DAPI (blue). Scale bars: 10 μm.

(D) Western blot analysis of pluripotency-related proteins in WT and *Gas5* KO ESCs. Bar plots show quantifications of OCT4, NANOG and SOX2 intensity normalized to α-Tubulin.

(E) Immunofluorescence staining of H3K9me3 (green) in MEFs treated with control siNC and Gas5-targeting siRNA (siGas5) at 0, 12-, 24-, 36-, and 48-hours post-transfection. Nuclei were counterstained with DAPI (blue). Scale bars: 10 μm. Bar plots show quantifications of H3K9me3 foci per cell. n = 155 (WT) and 155 (siGas5) cells pooled across three independent experiments.

(F) Western blot analysis of H3K4me1, H3K4me2, H3K4me3, H3K9me1, H3K9me2, H3K27me3 and H4K14ac in WT and *Gas5* KO ESCs. α-Tubulin was used as a loading control. Bar plots show quantifications of H3K4me1, H3K4me2, H3K4me3, H3K9me1, H3K9me2, H3K27me3 and H4K14ac intensity normalized to α-Tubulin.

(G) Western blot analysis of SUV39H1, SUV39H2 and SETDB1 in WT and *Gas5* KO ESCs. Bar plots show quantifications of SUV39H1, SUV39H2 and SETDB1 intensity normalized to α-Tubulin.

(H) Western blot analysis of KDM4A/B/C/D in WT and *Gas5* KO ESCs. Bar plots show quantifications of KDM4A/B/C/D intensity normalized to α-Tubulin.

(I) Western blot analysis of KDM4A/B/C/D in WT, WT (P25A/K333A) and Mut (P25A/K333A) (I) ESCs. Bar plots show quantifications of KDM4A/B/C/D intensity normalized to GAPDH.

(J) Gene scatter plot of the SUV39H2 ChIP-seq in WT and *Gas5* KO ESCs. *Gas5* depletion (y-axis) was compared to control (x-axis) in terms of fold change (log scale) of genes in IP relative to Input. Three independent clones in each condition were used. Ten enriched genes in SUV39H2 ChIP-seq are labeled.

(K) Bioinformatic analysis of H3K9me3 ChIP-seq datasets showing the relative $\log_2$ enrichment differences of H3K9me3 occupancy across distinct genomic features. The horizontal bar charts represent the distribution shifts in *Gas5* KO versus WT ESCs (left),

as well as in SUV39H2 RNA-binding deficient mutants P25A (middle) and K333A (right) versus their respective WT controls.

(L) RT-qPCR analysis the transcriptional activity of Major Satellites and LINE-1 elements in both *Gas5* KO and Mut (P25A/K333A) ESCs. Transcript levels were normalized to Gapdh.

Data in A, D and F-L are shown as mean ± SEM. (n=3 independent experiments).

**Alt Text:** A twelve-panel supplementary figure (A-L) supporting the role of LncRNA Gas5 in maintaining heterochromatin integrity and SUV39H2 occupancy. Panel A and L displays a bar chart of qPCR analysis results. Panels B and C feature fluorescence microscopy images of cells. Panels D, F-I present Western blot bands alongside corresponding bar charts for protein quantification. Panel E pairs fluorescence microscopy images with a bar chart quantifying cellular foci. Panel J is a gene scatter plot comparing sequencing fold changes between different genetic conditions. Panel K is a series of horizontal bar charts illustrating genomic distribution shifts of H3K9me3.

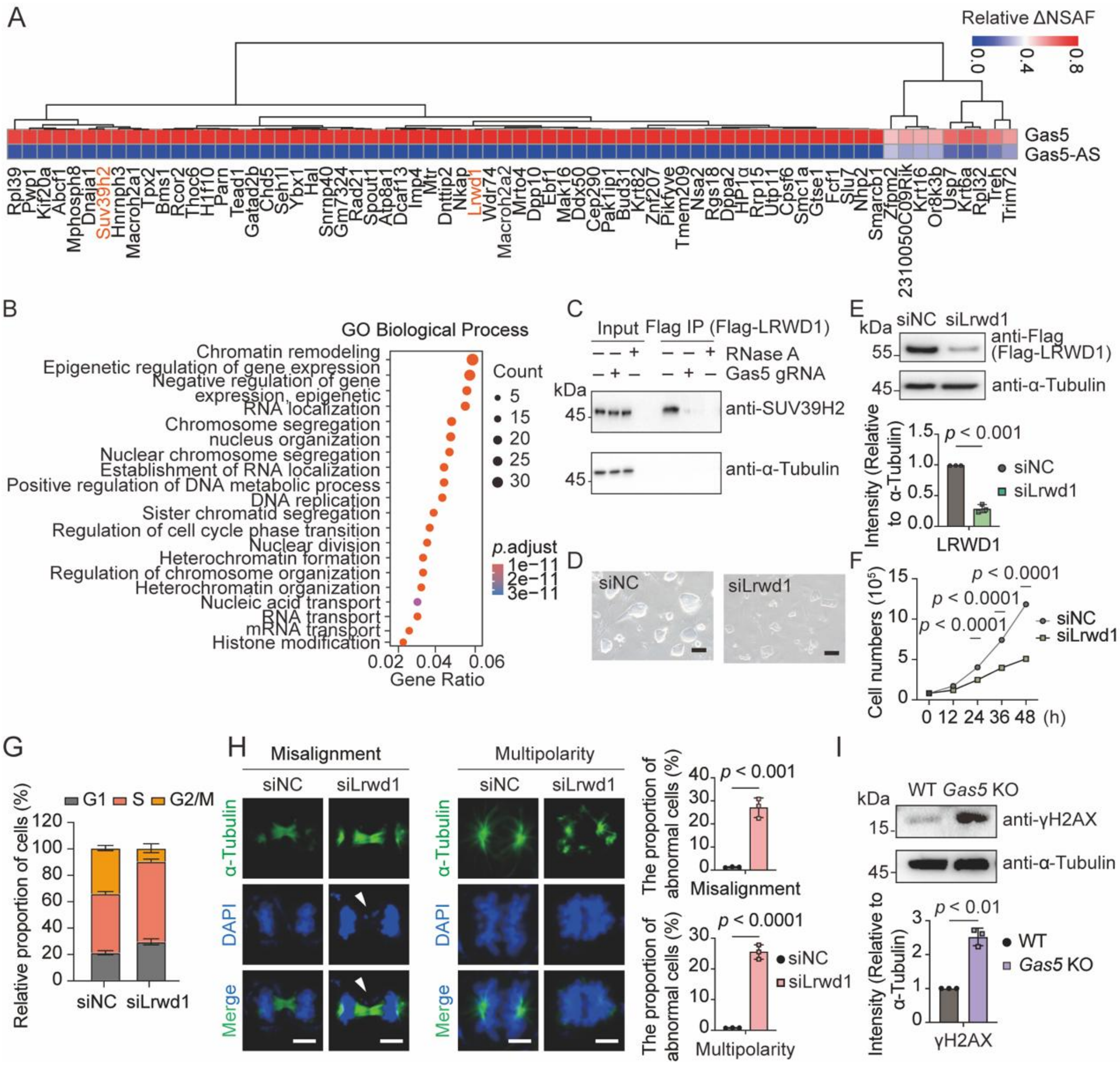


**Supplementary Figure 5. Gas5 scaffolds the LRWD1–SUV39H2 complex to safeguard genomic integrity.**

(A) Heat map of Gas5/Gas5 antisense-interacting proteins identified by immunoprecipitation mass spectrometry (IP-MS) in ESCs.

(B) Gene ontology (GO) analysis of Gas5-interacting proteins identified by IP-MS in ESCs.

(C) Co-immunoprecipitation (Co-IP) of endogenous LRWD1 with SUV39H2 in ESCs under three conditions: untreated, RNase A-treated, and *Gas5* KO. Bar plots show quantifications of SUV39H2 intensity normalized to Input.

(D) Bright-field images of siNC and siLrwd1 ESC colonies. Scale bar: 100 μm.

(E) Western blot analysis of LRWD1 in siNC and siLrwd1 ESCs. Bar plots show quantifications of LRWD1 intensity normalized to α-Tubulin.

(F) Proliferation curves of siNC and siLrwd1 ESCs measured by cell counting at 0, 12-, 24-, 36-, and 48-hours post-seeding.

(G) Cell cycle analysis of siNC and siLrwd1 ESCs. Histograms show the percentage of cells in G1, S, and G2/M phases.

(H) Representative images of mitotic errors, including alignment defects and multipolar spindles. Bar plots show the percentage of abnormal cells. n = 110 (WT), n = 110 (*Gas5* KO) cells pooled across three independent experiments. Scale bars, 5 μm.

(I) Western blot analysis of γH2AX in WT and *Gas5* KO ESCs. Bar plots show quantification of γH2AX intensity normalized to α-Tubulin.

Data in C, E, F, G and I are shown as mean ± SEM. (n=3 independent experiments).

Alt Text: A multi-panel figure (A–I) illustrating the biochemical and functional relationship between Gas5, LRWD1, and SUV39H2. Panels A and B display a colorful heat map of interacting proteins and a bar graph of GO enrichment terms. Panel C shows co-immunoprecipitation protein bands with a corresponding quantification bar graph. Panels D and H contain bright-field and fluorescence microscopy images of ESC colonies and mitotic defects (spindle abnormalities). Panels E and I are western blot images with quantification plots. Panel F presents a line graph showing cell proliferation rates over 48 hours, and Panel G shows cell cycle distribution histograms across G1, S, and G2/M phases.

**Supplemental Table 1: Oligonucleotide sequences used in this study.**

| Primer | Sequence (5' to 3') | Use |
|---|---|---|
| Gas5_qPCR_F | GGAGGATGAAGGCTTACGAGG | RT-qPCR |
| Gas5_qPCR_R | CAAGCAAGCCAGCCAAATGAA | RT-qPCR |
| Malat1_qPCR_F | TGCAGTGTGCCAATGTTTCG | RT-qPCR |
| Malat1_qPCR_R | GGCCAGCTGCAAACATTCAA | RT-qPCR |
| Neat1_qPCR_F | GCCTCAGACCCTCAGTTTCC | RT-qPCR |
| Neat1_qPCR_R | ATGCCATCCAGGTTCCAAGG | RT-qPCR |
| Rmrp_qPCR_F | GAAGGCCTGTTTCCTAGGCT | RT-qPCR |
| Rmrp_qPCR_R | TTCTTGGCGGGCTAACAGTG | RT-qPCR |
| Snhg12_qPCR_F | AGAGTCTGAGAAGACGGCCT | RT-qPCR |
| Snhg12_qPCR_R | GCTGCCTGGCAATGTACAAG | RT-qPCR |
| Oct4P4_qPCR_F | CCTCCTTTGGGGGAGACCTA | RT-qPCR |
| Oct4P4_qPCR_R | GGGAGAGGCCTTATCCTTGC | RT-qPCR |
| TERRA_qPCR_F | CGCAGAGCAAGACATGATGC | RT-qPCR |
| TERRA_qPCR_R | GCAGACCTCTCTGGAACCAC | RT-qPCR |
| Major Sat_qPCR_F | TGGAATATGGCGAGAAAACTG | ChIP qPCR |
| Major Sat_qPCR_R | AGGTCCTTCAGTGGGCATTT | ChIP qPCR |
| L1_promoter_qPCR_F | ACTGCGGTACATAGGGAAGC | ChIP qPCR |
| L1_promoter_qPCR_R | TGTGATCCACTCACCAGAGG | ChIP qPCR |
| L1_ORF1_qPCR_F | CACTCCCACCCCACCTAGT | ChIP qPCR |
| L1_ORF1_qPCR_R | TAACTCTTTAGCAGTGCTCTCCTGT | ChIP qPCR |
| L1_ORF2_qPCR_F | ACCTGGACGAAATGGACAAA | ChIP qPCR |
| L1_ORF2_qPCR_R | CATCTGGTCCTGGGCTTTT | ChIP qPCR |
| L1_3'UTR_qPCR_F | CCAGCAAACACAGAAGTGGA | ChIP qPCR |
| L1_3'UTR_qPCR_R | CCGACTAGGCCATCTTTTGA | ChIP qPCR |
| IAPEY3_qPCR-F | ACAGAGGAGGACAACTGCTC | ChIP qPCR |
| IAPEY3_qPCR_R | AACCTTACACAGGCAAAAGC | ChIP qPCR |
| ETnERV2_qPCR_F | ACAAATTCAGTATGGGCATC | ChIP qPCR |
| ETnERV2_qPCR_R | GGTACTGTTAAGACCCACA | ChIP qPCR |
| Gapdh_qPCR_F | AACTTTGGCATTGTGGAAGG | RT-qPCR, ChIP qPCR |
| Gapdh_qPCR_R | CACATTGGGGGTAGGAACAC | RT-qPCR, ChIP qPCR |
| Gas5 probe1 | ATGCCGCACAGCTCCGAAAG | RNA pull-down |
| Gas5 probe2 | CTGTGAGATCCCACAAGATG | RNA pull-down |
| Gas5 probe3 | TAAGCCTTCATCCTCCTTTG | RNA pull-down |
| Gas5 probe4 | TTATCCAGCTTCCTGACGAG | RNA pull-down |
| Gas5 probe5 | TTCTGGTCTTCTATTCTAGC | RNA pull-down |
| Gas5 probe6 | TTTCAAACTGTCTATCCAGC | RNA pull-down |
| Gas5 probe7 | AAGCAAGCCAGCCAAATGAA | RNA pull-down |
| Gas5 probe8 | ACTTGAGGTGACCCATTAAT | RNA pull-down |

| | | |
|---|---|---|
| Gas5 probe9 | CCGGAAATTCAGAATGACCA | RNA pull-down |
| Gas5 probe10 | AACAAAGGTGCAGTCACTGC | RNA pull-down |
| lacZ probe1 | CCAGTGAATCCGTAATCATG | RNA pull-down |
| lacZ probe2 | TCACGACGTTGTAAAACGAC | RNA pull-down |
| lacZ probe3 | ATTAAGTTGGGTAACGCCAG | RNA pull-down |
| lacZ probe4 | AGGTTACGTTGGTGTAGATG | RNA pull-down |
| lacZ probe5 | AATGTGAGCGAGTAACAACC | RNA pull-down |
| lacZ probe6 | GTAGCCAGCTTTCATCAACA | RNA pull-down |
| lacZ probe7 | AATAATTCGCGTCTGGCCTT | RNA pull-down |
| lacZ probe8 | AGATGAAACGCCGAGTTAAC | RNA pull-down |
| lacZ probe9 | AATTCAGACGGCAAACGACT | RNA pull-down |
| lacZ probe10 | TTTCTCCGGCGCGTAAAAAT | RNA pull-down |
| Gas5_gRNA_F | AGCTGTGCGGCATTCTGAGC | Knockout |
| Gas5_gRNA_R | GCTCAGAATGCCGCACAGCT | Knockout |
| Suv39h2_gRNA_F | CACCGGCGGCGTCTCGCCGTTCGCC | Knockout |
| Suv39h2_gRNA_R | AAACGGCGAACGGCGAGACGCCGCC | Knockout |
| siGas5 | GCUGGAUAGACAGUUUGAATT | Knockdown |
| siLrwd1 | ACGGUCAGUGACAACCUCAAA | Knockdown |

## References


AMEMIYA, H. M., KUNDAJE, A. & BOYLE, A. P. 2019. The ENCODE Blacklist: Identification of Problematic Regions of the Genome. *Scientific Reports,* 9**,** 9354.

DOBIN, A., DAVIS, C. A., SCHLESINGER, F., DRENKOW, J., ZALESKI, C., JHA, S., BATUT, P., CHAISSON, M. & GINGERAS, T. R. 2013. STAR: ultrafast universal RNA-seq aligner. *Bioinformatics*, 29**,** 15-21.

KENT, W. J., ZWEIG, A. S., BARBER, G., HINRICHS, A. S. & KAROLCHIK, D. 2010. BigWig and BigBed: enabling browsing of large distributed datasets. *Bioinformatics,* 26**,** 2204-2207.

LANGMEAD, B. & SALZBERG, S. L. 2012. Fast gapped-read alignment with Bowtie 2. *Nature Methods,* 9**,** 357-359.

LI, H., HANDSAKER, B., WYSOKER, A., FENNELL, T., RUAN, J., HOMER, N., MARTH, G., ABECASIS, G., DURBIN, R. & PROC, G. P. D. 2009. The Sequence Alignment/Map format and SAMtools. *Bioinformatics,* 25**,** 2078-2079.

LIAO, Y., SMYTH, G. K. & SHI, W. 2014. featureCounts: an efficient general purpose program for assigning sequence reads to genomic features. *Bioinformatics,* 30**,** 923-930.

LOVE, M. I., HUBER, W. & ANDERS, S. 2014. Moderated estimation of fold change and dispersion for RNA-seq data with DESeq2. *Genome Biology,* 15**,** 550.

QUINLAN, A. R. & HALL, I. M. 2010. BEDTools: a flexible suite of utilities for comparing genomic features. *Bioinformatics,* 26**,** 841-842.

STRIMMER, K. 2008. fdrtool: a versatile R package for estimating local and tail area-based false discovery rates. *Bioinformatics,* 24**,** 1461-1462.

THORVALDSDÓTTIR, H., ROBINSON, J. T. & MESIROV, J. P. 2013. Integrative Genomics Viewer (IGV): high-performance genomics data visualization and exploration. *Briefings in Bioinformatics,* 14**,** 178-192.

TRAPNELL, C., WILLIAMS, B. A., PERTEA, G., MORTAZAVI, A., KWAN, G., VAN BAREN, M. J., SALZBERG, S. L., WOLD, B. J. & PACHTER, L. 2010. Transcript assembly and quantification by RNA-Seq reveals unannotated transcripts and isoform switching during cell differentiation. *Nature Biotechnology,* 28**,** 511-515.

YU, G. C., WANG, L. G. & HE, Q. Y. 2015. ChIPseeker: an R/Bioconductor package for ChIP peak annotation, comparison and visualization. *Bioinformatics*, 31**,** 2382-2383.

ZHANG, Y., LIU, T., MEYER, C. A., EECKHOUTE, J., JOHNSON, D. S., BERNSTEIN, B. E., NUSSBAUM, C., MYERS, R. M., BROWN, M., LI, W. & LIU, X. S. 2008. Model-based Analysis of ChIP-Seq (MACS). *Genome Biology,* 9**,** R137.
